# Orally Administered Functional Polyphenol‐Nanozyme‐Armored Probiotics for Enhanced Amelioration of Intestinal Inflammation and Microbiota Dysbiosis

**DOI:** 10.1002/advs.202411939

**Published:** 2025-03-11

**Authors:** Yong Zhu, Ziqu Fang, Jie Bai, Longhui Wang, Jiaqing Chen, Zehua Zhang, Qiang Wang, Weiwei Sheng, Xueyin Pan, Zhenyuan Gao, Dengqiu Xu, Pengkai Wu, Beicheng Sun

**Affiliations:** ^1^ Department of Hepatobiliary Surgery The First Affiliated Hospital of Anhui Medical University Hefei Anhui 230022 China; ^2^ MOE Innovation Center for Basic Research in Tumor Immunotherapy Hefei Anhui 230022 China; ^3^ Anhui Province Key Laboratory of Tumor Immune Microenvironment and Immunotherapy Hefei Anhui 230022 China; ^4^ Department of General Surgery The First Affiliated Hospital of Anhui Medical University Hefei 230022 China

**Keywords:** inflammatory bowel disease, nanozyme, polyphenol, probiotics

## Abstract

Maintaining microbiota balance and enhancing the antioxidant performance of nanozyme‐based probiotic systems are crucial for effective inflammatory bowel disease (IBD) therapy. Despite significant advancements, developing a green and safe coating technology that functionalizes probiotics with nanozymes while preserving the activity of both components remains a challenge. To address this, chitosan‐modified epigallocatechin gallate (EGCG‐CS, EC)is synthesized, leveraging the intrinsic adhesive and coordination properties of polyphenols to capture gold nanozymes (AuNPs), forming ECA complexes that enhance nanozyme activity. When coated onto *Escherichia coli* Nissle 1917 (EcN), the resulting ECA@EcN system effectively scavenged reactive oxygen species (ROS), improving probiotic viability and promoting colon accumulation. Mechanistically, ECA protected EcN by suppressing the activation of the Flagellar Assembly and Branched‐Chain Amino Acid Synthesis pathways, ultimately alleviating inflammation and modulating intestinal microbial communities to relieve IBD symptoms. Given the biocompatibility of its components and the environmentally friendly assembly approach, this polyphenol‐nanozyme‐armored probiotic system represents a promising platform for IBD treatment.

## Graphical abstract

This study presents a novel polyphenol‐nanozyme‐armored probiotic system (ECA@EcN) for enhanced treatment of inflammatory bowel disease (IBD). By integrating chitosan‐modified epigallocatechin gallate (EGCG‐CS) with gold nanozymes, the system effectively scavenges ROS, improves probiotic viability, and restores gut microbiota balance. The eco‐friendly coating strategy improves probiotic resilience and facilitates targeted colon delivery, presenting a promising therapeutic approach for IBD management.

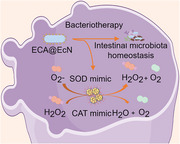

## 1 Introduction

Inflammatory bowel disease (IBD), encompassing ulcerative colitis (UC), Crohn's disease (CD), and undetermined colitis (IC), is a chronic and incurable condition that significantly impacts patients' quality of life.^[^ ^1^ ^]^ Although the exact pathogenesis of IBD remains complex and incompletely understood, key contributors include impaired intestinal barrier function, gut microbiota dysbiosis, and excessive immune responses driven by elevated reactive oxygen species (ROS) levels.^[^ ^2^ ^]^ Current clinical therapies primarily focus on anti‐inflammatory drugs and immunosuppressants, such as corticosteroids and tumor necrosis factor antagonists, to alleviate inflammation and manage symptoms. However, these treatments often exhibit limited long‐term efficacy and can induce severe adverse effects, restricting their broader clinical application.^[^ ^3^ ^]^

Probiotic‐based therapies have emerged as promising alternatives for restoring intestinal mucosa, suppressing pathogenic bacteria, and maintaining microbiota balance.^[^ ^4^ ^]^ Despite their potential, probiotics face significant challenges due to their susceptibility to ROS‐induced damage, as they lack critical antioxidant enzymes such as superoxide dismutase (SOD) and catalase (CAT).^[^ ^5^ ^]^ Moreover, their therapeutic efficacy is hindered by poor stability during oral administration. Exposure to harsh gastric acids and gastrointestinal stresses often results in substantial viability loss, reducing their concentration below therapeutic thresholds and limiting their effectiveness in clinical applications.^[^ ^5^, ^6^ ^]^

To overcome these challenges, nanozymes have gained attention as artificial enzymes that mimic the antioxidant activity of natural enzymes such as SOD and CAT. These materials effectively regulate ROS levels under inflammatory conditions, offering a potential solution for probiotic protection in the gastrointestinal tract.^[^ ^7^ ^]^ Polyphenols, renowned for their antioxidant and anti‐inflammatory properties, have been utilized to construct metal‐polyphenol networks (MPNs) for probiotic encapsulation.^[^ ^8^ ^]^ As a type of MPN, the polyphenol‐nanozyme system holds significant potential for probiotic applications. However, its therapeutic application in IBD remains underexplored, primarily due to the instability of metal‐phenolic structures under acidic gastrointestinal conditions.^[^ ^9^ ^]^

Encapsulation of probiotics within biopolymeric matrices presents another effective strategy for targeted gastrointestinal delivery.^[^ ^10^ ^]^ Chitosan (CS), a natural polysaccharide known for its excellent hydrophilicity, biocompatibility, and biodegradability, has been extensively used in both food and pharmaceutical applications.^[^ ^11^ ^]^ Its amino‐rich structure facilitates strong electrostatic interactions, enhancing adhesion to cells and nanoparticles, which makes it an ideal candidate for gastrointestinal drug delivery.^[^ ^12^ ^]^ Integrating CS into metal‐polyphenol‐based probiotic coating systems has the potential to overcome the limitations of MPN by enhancing probiotic stability, improving targeting precision, and boosting functional performance in therapeutic applications.

**Figure 1:**
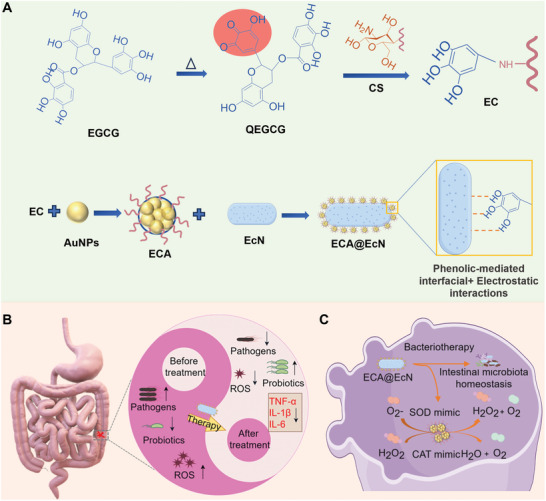
Schematic illustration of the construction of ECA@EcN and its mechanism for IBD treatment. A) ECA@EcN was constructed through phenolic‐metal coordination‐mediated interfacial and electrostatic interactions. B) Mechanism of ECA@EcN in modulating inflammatory responses and restoring the balance of intestinal flora. (C) ECA@EcN can mimic SOD and CAT antioxidant enzymes and regulate the intestinal flora.

In this study, we propose a green polyphenol‐armored strategy that utilizes the coordination properties of polyphenols to link nanozymes and probiotics through phenolic‐metal interactions. Epigallocatechin‐3‐gallate (EGCG), a phenolic compound derived from green tea, possesses potent antioxidative, anti‐inflammatory, and antimicrobial properties.^[^ ^13^ ^]^ EGCG has shown therapeutic potential in IBD by scavenging ROS and promoting beneficial bacterial abundance.^[^ ^14^ ^]^ In this study, CS was incorporated into the EGCG system to synthesize EGCG‐CS (EC) through a simple mixing method, aiming to enhance nanozyme activity and improve stability. Gold nanoparticles (AuNPs), selected for their robust antioxidant enzyme‐mimicking capabilities, were coordinated with EC to form ECA complexes, which were subsequently used to coat *Escherichia coli* Nissle 1917 (EcN) through phenolic‐mediated interfacial and electrostatic interactions (**Figure** **1**A).

Mechanistic investigations revealed that the ECA@EcN system effectively reduced ROS levels, suppressed pro‐inflammatory cytokine production, and restored intestinal barrier integrity by inhibiting the Flagellar Assembly and Branched‐Chain Amino Acid Synthesis pathways. Furthermore, oral administration of ECA@EcN led to increased gut microbiota richness and diversity, ultimately yielding favorable therapeutic outcomes in IBD (Figure 1B,C). This study establishes a practical and promising platform for effective IBD remission by harnessing the synergistic interactions between polyphenols, nanozymes, and probiotics, thereby expanding the potential applications of polyphenol‐nanozyme‐armored probiotics in clinical settings.

## 2 Results and Discussion

### 2.1 Fabrication and Characterization of ECA and ECA@EcN

Nanozymes have shown great potential in regulating redox homeostasis, particularly in the context of IBD treatment.^[^ ^5^, ^7^, ^15^ ^]^ In this study, we synthesized a range of nanozymes and evaluated their SOD‐like activities (Figure S1, Supporting Information). Among the different candidates, AuNPs exhibited the highest SOD‐like activity and were chosen for further investigation.

**Figure 2:**
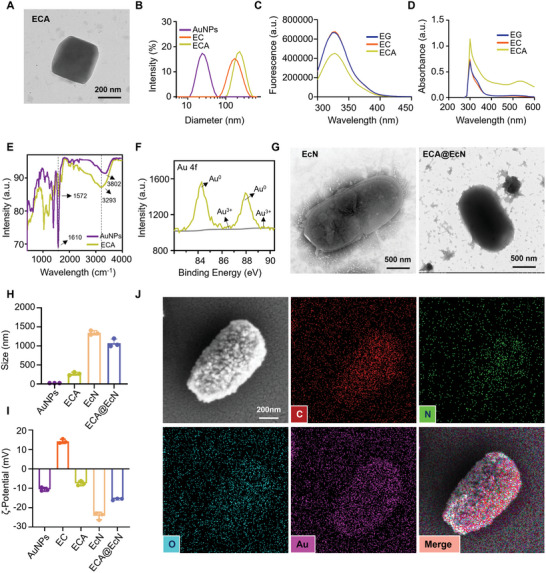
Characterization of ECA and ECA@EcN. A) Representative TEM images of ECA. B) Size distributions of AuNPs, EC, and ECA. C) Fluorescence spectra and D) UV spectrum of EGCG, EC, and ECA. E) FT‐IR spectra of AuNPs and ECA. F) High‐resolution XPS spectra of ECA. G) Representative TEM image of EcN and ECA@EcN. H,I) Particle sizes and zeta potentials of AuNPs, ECA, EcN, and ECA@EcN (n = 3). J) SEM and element mapping images of ECA@EcN. Data are presented as the means ± SD. Statistical analysis was performed using one‐way ANOVA. *p < 0.05; ns, not significant.

To construct the ECA system, EC was synthesized through a Schiff base reaction between oxidized EGCG (EGCG quinone, QEGCG) and the amino groups of CS. The structure of EC was confirmed by proton nuclear magnetic resonance spectroscopy (¹H‐NMR) (Figure S2, Supporting Information). AuNPs were then synthesized and coordinated with EC through the inherent affinity of EGCG for metal ions, resulting in the formation of the ECA complex. Transmission electron microscopy (TEM) images revealed the uniform size and irregular morphology of the synthesized ECA (**Figure** **2**A). Dynamic light scattering (DLS) analysis showed that the average diameters of AuNPs, EC, and ECA were ≈22.5, 162.5, and 224.4 nm, respectively (Figure 2B).

The successful formation of ECA was further confirmed by fluorescence and ultraviolet (UV) spectroscopy. Specifically, a reduction in fluorescence intensity at the C═O chromophore and an increase in surface plasmon resonance (SPR) absorption were observed, confirming the formation of the metal‐phenolic complex (Figure 2C,D). Fourier‐transform infrared (FT‐IR) spectroscopy revealed a shift in the ‐OH absorption peak from 3802 to 3293 cm⁻¹, and a red shift in the C═O peak from 1572 to 1610 cm⁻¹, suggesting the involvement of hydroxyl and carboxyl groups in the stabilization of ECA (Figure 2E). X‐ray photoelectron spectroscopy (XPS) identified characteristic peaks for O 1s, C 1s, and Au 4f (Figure S3A, Supporting Information). The peaks at 83.4 and 87.1 eV in the Au 4f spectrum corresponded to Au^3^⁺ and Au⁰ oxidation states, respectively, confirming the formation of Au‐O bonds, a crucial step in the synthesis of ECA (Figure 2F). UV–vis spectroscopy showed a ligand‐to‐metal charge‐transfer band ≈ 530 nm, consistent with bis‐state metal‐phenolic coordination (Figure S3B, Supporting Information). X‐ray diffraction (XRD) analysis revealed broad peaks at 38.3°, 44.4°, 64.7°, and 77.6°, corresponding to the (111), (200), (220), and (311) planes of the Au lattice (JCPDS Card No. 04–0784) (Figure S3C, Supporting Information).

EcN was chosen for this study due to its proven antibacterial, anti‐inflammatory, and microbiota‐modulating properties in gastrointestinal disorders.^[^ ^12^, ^16^ ^]^ To achieve targeted delivery to inflamed colonic regions, ECA was adsorbed onto the surface of EcN to form a compact protective coating. The color change of the ECA@EcN complex from white (EcN) to black upon ECA deposition visually confirmed the successful coating (Figure S4, Supporting Information). DLS analysis revealed that the average size of ECA@EcN decreased from 1342.7 ± 57.1 nm to 1073 ± 106.9 nm, while the zeta potential decreased from −24.3 ± 1.9 mV to −15.7 ± 0.5 mV. TEM confirmed the formation of a stable ECA shell around EcN (Figure 2G–I). This reduction in size was likely due to mechanical constraints imposed by hydrogen bonding and electrostatic interactions, which compressed the soft cell walls of EcN (Figure S5A–C, Supporting Information).

The effects of the coatings, including EA, CS, and ECA, on EcN viability were assessed by monitoring growth curves over a 12‐h period, based on optical density at 600 nm (OD600). The growth rates of naked EcN, EA@EcN, and ECA@EcN were comparable (Figure S5D, Supporting Information). However, the CS coating had a moderate impact on EcN growth due to increased cell wall permeability caused by electrostatic interactions, which led to cellular leakage and membrane disruption.^[^ ^17^ ^]^ EGCG mitigated this effect by forming hydrogen and covalent bonds with CS, which reduced its positive charge and attenuated its stimulatory effects on EcN.^[^ ^18^ ^]^ As a result, the ECA coating did not significantly impair EcN viability.

Laser scanning confocal microscopy (LSCM) further validated the presence of the ECA layer on EcN. This was demonstrated by the colocalization of Cy5‐labeled ECA and Hoechst 33342‐labeled EcN fluorescence (Figure S6, Supporting Information). Scanning electron microscopy (SEM) combined with energy‐dispersive spectroscopy (EDS) confirmed that the Au element, representing the ECA complex, was uniformly adsorbed onto the surface of EcN (Figure 2J). These findings collectively demonstrate the successful fabrication of the ECA@EcN complex.

### 2.2 Antioxidant and Anti‐Inflammatory Activities In Vitro

Excessive ROS plays a critical role in the pathogenesis of IBD.^[^ ^19^ ^]^ The polyphenolic structure of EGCG has long been recognized for its potent antioxidant properties. In this study, the antioxidant and anti‐inflammatory potential of the ECA composite, a nanoparticle system composed of EGCG‐CS conjugates and AuNPs, was assessed through various in vitro assays.

To evaluate the radical‐scavenging capabilities of ECA, both DPPH and ABTS assays were utilized. These tests confirmed that EGCG conjugated with CS demonstrated excellent antioxidant properties,^[^ ^20^ ^]^ and that the addition of AuNPs did not hinder the antioxidant ability of EGCG (Figure S7, Supporting Information). Further enzyme‐mimicking assays targeting superoxide anions (•O₂⁻), hydrogen peroxide (H₂O₂), and hydroxyl radicals (•OH) illustrated the ROS‐scavenging capacity of ECA. Notably, AuNPs displayed both SOD‐like and peroxidase (POD)‐like activities.^[^ ^21^ ^]^ However, POD activity, which leads to the formation of hydroxyl radicals, can be harmful in biological systems.^[^ ^22^ ^]^ The integration of CS with AuNPs allowed the transformation of POD‐like activity intoCAT‐like activity,^[^ ^21^ ^]^ which was beneficial in neutralizing oxidative stress.

**Figure 3:**
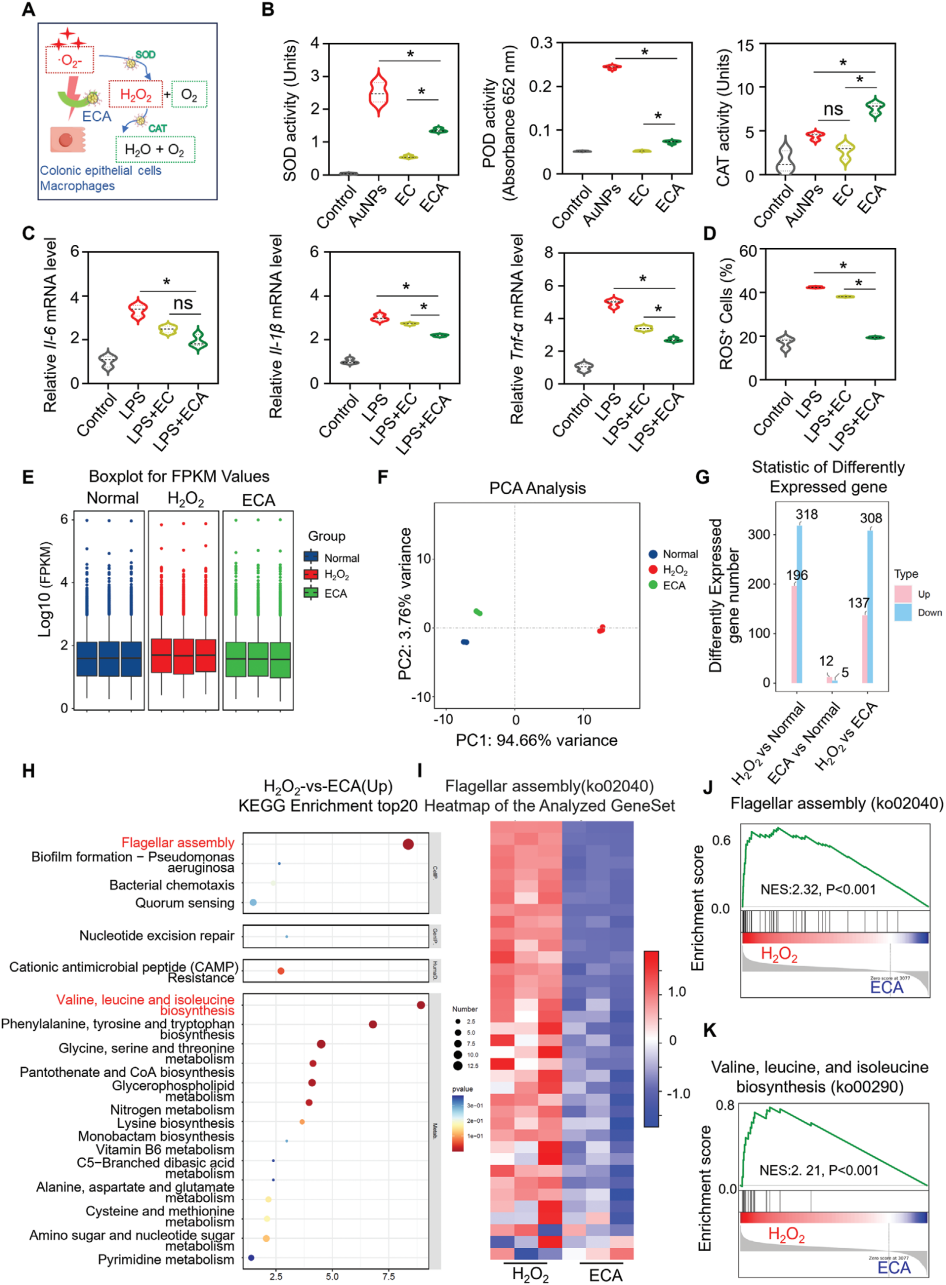
Antioxidant activities, anti‐inflammatory effects, and RNA sequencing (RNA‐seq) analysis of ECA in protecting EcN. A) Illustration of ECA's ROS scavenging mechanism as an SOD and CAT antioxidant mimic. B) SOD‐, POD‐, and CAT‐like activities of ECA. C) Relative mRNA expression levels of Il‐6, Il‐1β, and Tnf‐α in RAW 264.7 cells stimulated with LPS for 24 h. D) Quantitative analysis of ROS production in RAW 264.7 cells under various treatment conditions were performed using flow cytometry. E) FPKM (fragments per kilobase of transcript per million fragments mapped) analysis. F) Principal component analysis (PCA). G) Statistics of differentially expressed genes (DEGs). H) KEGG pathway classification of upregulated genes. I) Heatmaps of RNA‐seq data for Flagellar assembly genes downregulated in ECA‐coated EcN. J,K) GSEA analysis for Flagellar assembly genes and Valine, Leucine, and Isoleucine Biosynthesis genes in H_2_O_2_‐treated versus ECA‐treated conditions. NES, normalized enrichment score. Data are presented as the means ± SD (n = 3). Statistical analysis was performed using one‐way ANOVA. *p < 0.05; ns, not significant.

This transformation was attributed to the amine‐rich structure of CS, which effectively scavenged highly reactive •OH radicals generated during the Fenton reaction through amine oxidation (Figure S8, Supporting Information).^[^ ^21^, ^23^ ^]^ ECA demonstrated superior H₂O₂ elimination compared to individual components such as AuNPs or the EC conjugate alone (**Figure** **3**B). The SOD‐like activity of ECA was pH‐dependent, with its activity significantly increasing in acidic environments (Figure S9A, Supporting Information), likely due to the protonation of citrate groups on the AuNP surface. Furthermore, ECA exhibited high CAT‐like activity across the pH range of 2.1–7.4 (Figure S9B, Supporting Information).

Macrophages play a pivotal role in the progression of IBD by producing inflammatory cytokines such as IL‐6, IL‐1β, and TNF‐α, which also lead to excessive ROS production.^[^ ^24^ ^]^ This creates a feedback loop exacerbating the inflammatory response.^[^ ^25^ ^]^ In the study, RAW264.7 macrophages were stimulated with lipopolysaccharide (LPS) and interferon‐gamma (IFN‐γ), which led to increased levels of these pro‐inflammatory cytokines. The treatment of these macrophages with ECA significantly reduced the expression of these cytokines (Figure 3C), demonstrating the superior anti‐inflammatory properties of ECA compared to EGCG‐CS alone.

ROS levels in macrophages were also measured using the DCFH‐DA probe and flow cytometry. As shown in Figures 3D and S10A (Supporting Information), ROS levels in LPS‐stimulated macrophages increased to 43%, but decreased to 38.3% and 19.4% following EC and ECA treatments, respectively. ECA also effectively protected human colonic epithelial cells (SW480) from H₂O₂‐induced oxidative damage, as indicated by the restoration of cell viability from 40.9% to 97.3% following ECA treatment (Figure S10B, Supporting Information).

As mitochondria are the main producers of intracellular ROS, the researchers used the MitoSOX probe to specifically detect mitochondrial superoxide anions (•O₂⁻).^[^ ^26^ ^]^ ECA treatment significantly reduced mitochondrial ROS levels in LPS‐stimulated macrophages and human colonic epithelial cells (HT29). The results indicated that ECA was highly effective in mitigating mitochondrial oxidative damage, further supporting its potential as a therapeutic agent for IBD (Figure S11, Supporting Information). Notably, ECA exhibited negligible hemolysis, indicating its excellent biocompatibility, which is crucial for its application in vivo, especially in the blood circulation system (Figure S12, Supporting Information).

### 2.3 Mechanism of ECA in Protecting EcN

ECA's protective mechanism was further investigated using transcriptome sequencing on *E. coli* Nissle (EcN), a probiotic strain beneficial for gastrointestinal health. EcN was exposed to H₂O₂‐induced oxidative stress to simulate an inflammatory condition resembling IBD.

Gene expression analysis revealed consistent expression profiles within each experimental group (Figure 3E), and principal component analysis (PCA) confirmed the reliability of the transcriptomic data (Figure 3F). The protective effect of ECA was demonstrated by the significant reduction in differentially expressed genes (DEGs) in ECA‐coated EcN compared to uncoated EcN exposed to oxidative stress (Figure 3G; Figure S13A, Supporting Information). KEGG and Gene Ontology (GO) analyses revealed that oxidative stress‐induced gene expression related to flagellar assembly and branched‐chain amino acid synthesis was downregulated in ECA‐coated EcN (Figure 3H,I; Figure S13B–D, Supporting Information).^[^ ^27^ ^]^ This was indicative of a reduction in stress responses and improved cellular integrity under oxidative conditions. These findings were corroborated by Gene Set Enrichment Analysis (GSEA), which showed that ROS‐responsive genes were normalized in ECA‐coated EcN (Figure 3J,K; Figure S13E,F, Supporting Information).

This suggests that ECA not only prevents oxidative damage but also plays a crucial role in maintaining the intestinal barrier function and promoting a healthier gut microbiome under inflammatory conditions, which are often associated with diseases like IBD.

### 2.4 Resistance Against Gastrointestinal Assaults and Biodistribution of ECA@EcN

**Figure 4:**
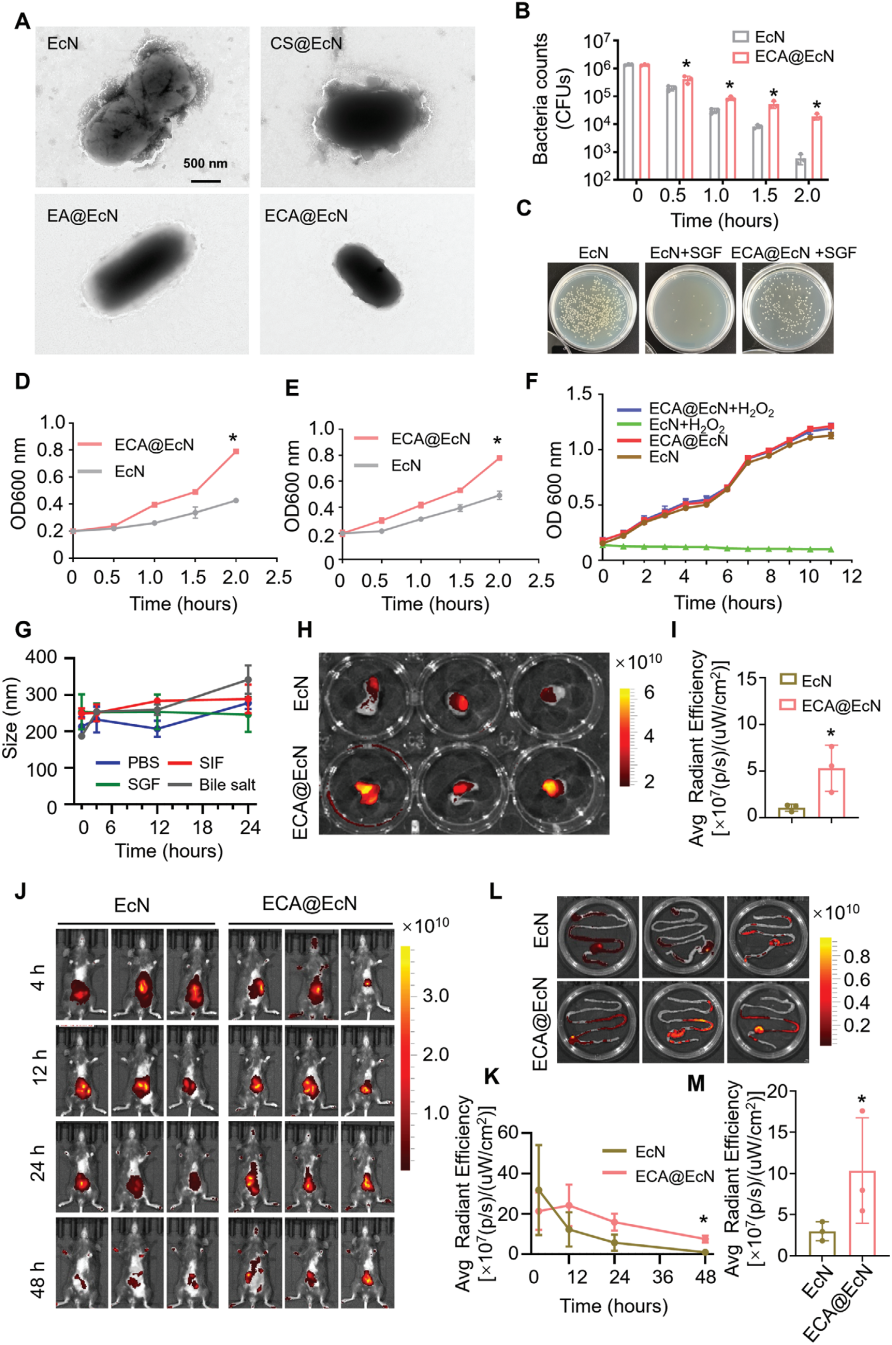
Resistance of ECA@EcN against gastrointestinal challenges and its biodistribution. A) TEM images of EcN, CS@EcN, EA@EcN, and ECA@EcN after incubation in SGF for 0.5 h at 37 °C. B) Survivals of EcN and ECA@EcN after exposure to SGF. C) Photographs of bacterial colonies of EcN and ECA@EcN after 0.5 h incubation in SGF. D,E) Survivals of EcN and ECA@EcN after exposure to SIF (pH 6.8) (D) and bile salts supplemented with trypsin (E) (n = 3). F) Growth curve of EcN and ECA@EcN in LB medium with or without H_2_O_2_ (100 µM) at 37 °C (n = 5). G) Fluorescence images of ex vivo intestines incubated with Cy5‐EcN and ECA@Cy5‐EcN. H) Quantification of chemiluminescence signals of EcN and ECA@EcN (n = 3). I) Chemiluminescence images of mice administered Cy5‐labeled bacterial formulations at different time points. J) Chemiluminescence decay curve of bioluminescence intensities analyzed over time. K) Chemiluminescence images of mouse intestinal tracts at 48 h post‐administration. L) ROI analysis of intestinal tract chemiluminescence intensities at 48 h (n = 3). Data are presented as the means ± SD. Statistical analysis was conducted using unpaired two‐tailed Student's *t*‐test (two groups) or one‐way ANOVA with Tukey's multiple comparisons (three groups). *p < 0.05.

Encapsulation with a modified prebiotic‐based ‘shield’ significantly enhances the ability of probiotics to withstand environmental stresses, including those encountered in the gastrointestinal tract.^[^ ^5^, ^6^, ^16^, ^28^ ^]^ Probiotics encounter a range of harsh conditions during transit through the gastrointestinal (GI) tract, such as gastric acid, bile salts, and inflammation, which can destabilize and deactivate EcN.^[^ ^5^ ^]^ To address these challenges, we encapsulated EcN with a modified prebiotic‐based ‘shield’ to enhance its resilience. To evaluate the protective effects of the ECA coating against gastric stress, we exposed both EcN and ECA@EcN to simulated gastric fluid (SGF, pH 1.5) containing 0.32% pepsin. Significantly, transmission electron microscopy (TEM) analysis showed that while the morphology of uncoated EcN was disrupted after 0.5 h in SGF, ECA‐coated EcN retained its structural integrity (**Figure** **4**A). The survival rates of ECA@EcN were significantly higher than those of uncoated EcN at 0.5, 1, 1.5, and 2 h of incubation (Figure 4B,C). After 2 h, more than 1,400 viable probiotics persisted in the ECA@EcN group, while nearly all uncoated EcN cells were eliminated. These findings suggest that the superior resistance of ECA@EcN to SGF may be attributed to the protective effects of hydrogen bonding and electrostatic interactions, which help repel stomach acids and maintain EcN's structural integrity (Figure S14, Supporting Information).

The viability of ECA@EcN was further evaluated in simulated intestinal fluid (SIF) and bile salts, conditions critical for survival during probiotic transit to the inflamed colon.^[^ ^29^ ^]^ Growth patterns of EcN and ECA@EcN were monitored every 30 min for 2 h using a microplate reader. ECA@EcN demonstrated significantly improved tolerance to both SIF (Figure 4D) and bile salts (Figure 4E) compared to uncoated EcN.

To examine whether the ECA coating affected EcN growth and proliferation, ECA@EcN was incubated in LB medium, and growth curves were tracked over 11 h. Comparable growth rates between EcN and ECA@EcN were observed, indicating minimal impact of the ECA coating on EcN's normal growth. Additionally, when exposed to H₂O₂, ECA@EcN showed significantly faster growth rates compared to uncoated EcN, indicating that the ECA coating plays a key cytoprotective role by shielding EcN from reactive oxygen species (ROS)‐induced damage (Figure 4F). To confirm the stability of the ECA coating, we analyzed changes in particle size after 24 h of incubation in PBS, SGF, SIF, and bile salts. No significant size changes were observed in any medium, further supporting the robust protective properties of the ECA coating (Figure 4G).

The protective effects of ECA against external stress conditions were further evaluated using PI staining assays. Exposure to H₂O₂, a toluene‐water interface, or an acidic solution (pH 4) resulted in extensive death of uncoated EcN cells.^[^ ^30^ ^]^ However, ECA‐coated EcN demonstrated significantly reduced cell death compared to uncoated cells, with the ECA coating providing superior protection relative to CS or EA alone (Figure S15, Supporting Information).

Recent studies have indicated that artificial enzyme‐modified probiotics effectively adhere to the mucosal barrier.^[^ ^5^ ^]^ Mucoadhesive properties of ECA@EcN were assessed using Cy5‐labeled EcN incubated in freshly isolated mouse intestines. Fluorescence intensity in the ECA@EcN group was significantly higher than that in the uncoated EcN group, indicating enhanced adhesion of ECA@EcN to intestinal mucosa (Figure 4H,I). Building on these findings, intestinal retention of ECA@EcN was evaluated in vivo. Mice treated with ECA@EcN exhibited prolonged intestinal retention compared to mice treated with uncoated EcN. Fluorescence signals from uncoated EcN diminished significantly within 24 h of administration, whereas ECA@EcN retained strong fluorescence signals even after 48 h (Figure 4J,K). IVIS imaging of intestinal tissues collected post‐euthanasia confirmed that fluorescence intensity in the ECA@EcN‐treated group was 3.5‐fold higher than that in the EcN‐treated group (Figure 4L,M). Long‐term retention of probiotics in the intestine is critical for enhancing their therapeutic efficacy, especially after passing through the upper gastrointestinal tract (GIT).^[^ ^31^ ^]^ In this study, we observed that ECA@EcN exhibited significantly prolonged retention in the intestine, which could potentially enhance its therapeutic effects.

Biodistribution studies further demonstrated the enhanced adhesive capacity of ECA@EcN. When one‐tenth of the previous dose was administered, ECA@EcN displayed a 4.3‐fold increase in adhesion compared to uncoated EcN (Figure S16, Supporting Information). In a dextran sulfate sodium (DSS)‐induced colitis model, ECA@EcN accumulated preferentially in inflamed colonic tissue, as evidenced by higher fluorescence intensity compared to normal colon tissue (Figure S17A–C, Supporting Information). Fluorescence signals from Cy5‐labeled ECA@EcN were absent in major organs, including the heart, liver, spleen, lungs, and kidneys, confirming the intestinal specificity of the ECA coating (Figure S17D, Supporting Information).

Collectively, these results demonstrate that the ECA coating significantly enhances EcN's resistance to gastrointestinal stresses, prolongs its intestinal retention, and improves mucoadhesive properties, underscoring its potential for targeted therapeutic applications in IBD.

### 2.5 Prophylactic Efficacy of ECA@EcN in DSS‐Induced Colitis

**Figure 5:**
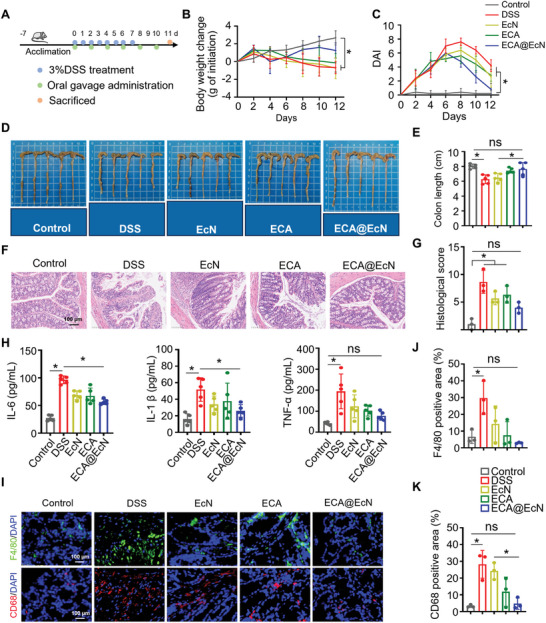
Prophylactic efficacy of ECA@EcN in a DSS‐induced mouse colitis model. A) Schematic representation of the administration schedule in a DSS‐induced acute colitis model. B) Body weight changes of mice subjected to different treatments (n = 5). C) Disease activity index (DAI) changes during treatment (n = 5). D,E) Colon tissue images and corresponding quantified colon lengths after various treatments (n = 5). F) Representative H&E‐stained images of colon tissues and G) histopathological scoring based on H&E images (n = 3). Scale bar: 100 µm. H) Serum levels of TNF‐α, IL‐6, and IL‐1β measured by ELISA on day 12 (n = 5). I) Representative immunofluorescence images of F4/80‐ and CD68‐positive cells in colon tissue sections from different groups (n = 3). J,K) Relative fluorescence intensities of F4/80‐ and CD68‐positive cells (n = 3). Scale bar: 100 µm. All data are presented as the means ± SD. Statistical analysis was performed using one‐way ANOVA. *p < 0.05; ns, not significant.

Building on the promising in vitro results, we further evaluated the prophylactic efficacy of ECA@EcN in vivo using a DSS‐induced colitis model. Colitis was induced in C57BL/6 mice by administering DSS for one week (**Figure** **5**A). The mice were then randomly assigned to five groups: Control, DSS, EcN, ECA, and ECA@EcN. Each formulation was administered orally every two days for a total of six doses, and the mice were euthanized on day 12.^[^ ^6^ ^]^ Prophylactic efficacy was then assessed using several parameters: body weight, disease activity index (DAI), colon length, histological evaluation, and levels of inflammatory cytokines.

As illustrated in Figure 5B,C, the control group exhibited continuous weight gain and stable DAI scores, indicating normal growth and no signs of inflammation. In contrast, the DSS‐treated group showed significant weight loss, accompanied by steadily increasing DAI scores starting on day 4. Notably, mice treated with ECA@EcN began to recover weight starting on day 6, and their DAI scores decreased, while the other groups continued to lose weight. Colonic inflammation often leads to colon shortening due to chronic tissue damage.^[^ ^5^ ^]^ As shown in Figure 5D,E, the DSS‐treated group exhibited a 21.7% reduction in colon length compared to the control group. In contrast, mice treated with ECA@EcN showed only a 4.7% reduction in colon length, emphasizing its superior prophylactic efficacy in protecting against colitis‐induced tissue damage.

Histopathological analysis was performed using a scoring system to assess the severity of inflammation, depth of injury, crypt damage, and tissue involvement.^[^ ^32^ ^]^ While the DSS‐treated groups exhibited significantly higher damage scores compared to the control group, the ECA@EcN group showed minimal histological changes, suggesting that it effectively mitigated epithelial damage and crypt erosion caused by DSS‐induced colitis (Figure 5F,G). To explore the anti‐inflammatory mechanism of ECA@EcN, we measured serum levels of proinflammatory cytokines (IL‐6, IL‐1β, and TNF‐α) using ELISA. ECA@EcN significantly reduced the levels of these cytokines compared to the DSS‐treated group, confirming its potent anti‐inflammatory activity (Figure 5H).

The inflammatory microenvironment in IBD disrupts immune homeostasis and is characterized by excessive macrophage infiltration in inflamed colonic tissues.^[^ ^33^ ^]^ Immunofluorescence staining for F4/80 and CD68, established markers of macrophages in mice,^[^ ^34^ ^]^ revealed substantial macrophage accumulation in DSS‐induced colonic lesions. ECA@EcN treatment significantly reduced macrophage infiltration, restoring immune balance in the colon (Figure 5I–K).

To assess systemic safety, we measured liver toxicity markers, including ALT and aspartate aminotransferase (AST). Neither marker showed significant changes in any treatment group, suggesting that ECA@EcN is non‐toxic to the liver (Figure S18A,B, Supporting Information). Histopathological analysis of major organs showed no pathological abnormalities in any treatment group compared to healthy controls (Figure S18C, Supporting Information).

Together, these findings demonstrate the efficacy of ECA@EcN in reducing inflammation, mitigating macrophage infiltration, and protecting against DSS‐induced colitis, while maintaining systemic safety and organ health.

### 2.6 Therapeutic Efficacy of ECA@EcN in DSS‐Induced Colitis

**Figure 6:**
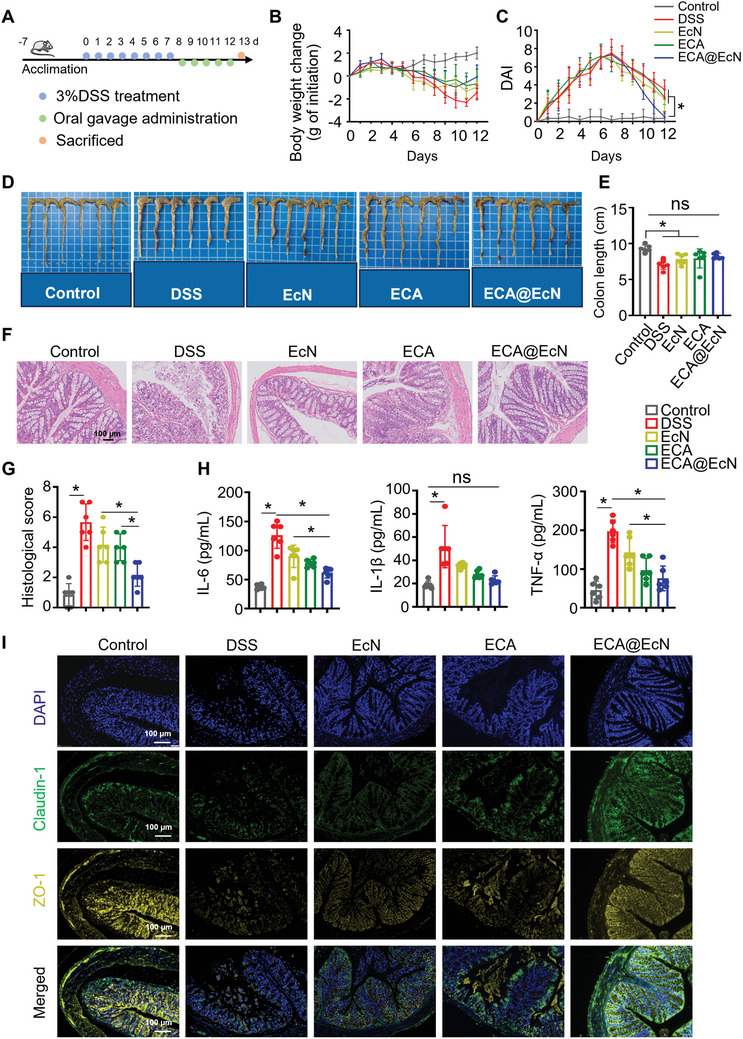
Therapeutic efficacy of ECA@EcN in a DSS‐induced mouse colitis model. A) Schematic representation of the therapeutic evaluation procedure for ECA@EcN in a DSS‐induced colitis model. B) Body weight variations in mice subjected to different treatments. C) Changes in disease activity index (DAI) during treatment. D,E) Colon tissue images and corresponding quantified colon lengths after various treatments. F,G) H&E‐stained histological images of colon tissues and corresponding histopathological scores. Scale bar: 100 µm. H) Serum levels of TNF‐α, IL‐6, and IL‐1β measured by ELISA on day 13. I) Representative immunofluorescence images of claudin‐1 (green) and ZO‐1 (yellow) with DAPI (blue) nuclear counterstaining. Scale bar: 100 µm. All data are presented as the means ± SD (n = 6). Statistical analysis was performed using one‐way ANOVA. *p < 0.05; ns, not significant.

The therapeutic efficacy of ECA@EcN in treating colitis was evaluated using a DSS‐induced colitis model, as illustrated in **Figure** **6**A. Mice were administered 3% DSS orally for seven days to establish the model.^[^ ^35^ ^]^ By day 7, all mice exhibited weight loss and elevated disease activity index (DAI) scores, confirming successful colitis induction. After establishing the colitis model, DSS administration was discontinued, and various formulations of EcN and ECA were subsequently administered orally for five consecutive days to evaluate their therapeutic effects. Therapeutic efficacy was assessed using parameters including body weight, DAI, colon length, histological analysis, and inflammatory cytokine levels.

As shown in Figure 6B,C, all treatment groups exhibited reduced weight loss and DAI compared to the DSS group after five days of treatment. Notably, the ECA@EcN group experienced significantly less weight loss and lower DAI scores than other treatment groups, indicating enhanced therapeutic efficacy, potentially due to its superior anti‐inflammatory and protective effects on gut barrier integrity (Figure S19, Supporting Information). Additionally, the colon length in the control group was significantly longer than in the DSS group. The colon length of mice treated with ECA@EcN showed no significant difference from that of the control group, highlighting its protective effect against colitis‐induced tissue damage (Figure 6D,E).

Histological examination of colonic tissues further demonstrated the effectiveness of ECA@EcN. The histopathological scores in the ECA@EcN‐treated group were significantly lower than those in all other DSS treatment groups, with reductions of 2.3‐fold, 1.7‐fold, and 1.6‐fold compared to the DSS, EcN, and ECA groups, respectively (Figure 6F,G). These results suggest that ECA@EcN significantly contributes to the repair and preservation of colonic tissue integrity.

These histological findings underscore the potential of ECA@EcN in maintaining colonic tissue integrity. To further investigate the underlying anti‐inflammatory mechanisms, serum cytokine levels were analyzed using ELISA. As expected, ECA@EcN treatment significantly decreased IL‐6, IL‐1β, and TNF‐α secretion, reinforcing its potent systemic anti‐inflammatory effects (Figure 6H).

Given that intestinal barrier disruption is a hallmark of colitis, we further examined the expression of tight junction proteins, Zonula occludens‐1 (ZO‐1) and claudin‐1, which are critical for epithelial integrity.^[^ ^35^ ^]^ DSS treatment markedly reduced their levels in colon tissues. However, ECA@EcN treatment significantly restored their expression, indicating its capacity to repair the intestinal epithelial barrier (Figure 6I)

Furthermore, the safety profile of ECA@EcN was rigorously evaluated through serum biochemical analysis and histological examination, confirming its non‐toxic nature (Figure S20, Supporting Information). Encouraged by these promising results, we further explored the prophylactic potential of ECA@EcN through an alternative delivery method using drinking water (Figure S21A, Supporting Information).

In this model, the ECA@EcN group exhibited significantly improved outcomes, including reduced weight loss, lower DAI scores, and longer colon lengths compared to the EcN or ECA groups (Figure S21B–E, Supporting Information), thereby highlighting its potential as a convenient and effective prophylactic intervention. Histological scoring and cytokine analysis further confirmed the superior efficacy of ECA@EcN in reducing inflammation and protecting colonic tissues (Figure S21F–H, Supporting Information). Importantly, no pathological changes were observed in major organs, reaffirming the safety of ECA@EcN (Figure S22, Supporting Information).

Collectively, these findings demonstrate the therapeutic potential of ECA@EcN in restoring intestinal homeostasis in a colitis model, attributed to its synergistic ROS‐scavenging and anti‐inflammatory properties. Future studies could explore its long‐term effects and potential clinical translation for colitis management.

### 2.7 Modulatory Effect of ECA@EcN on the Gut Microbiome

Disruptions in gut microbiota composition and their metabolites play a critical role in the onset and progression of IBD.^[^ ^36^ ^]^ In this context, probiotics have emerged as promising therapeutic agents due to their ability to modulate gut microbiota and confer health benefits to the host.^[^ ^5^, ^12^ ^]^ To assess the potential of ECA@EcN in restoring microbial balance, changes in gut microbiota composition were analyzed using high‐throughput sequencing of the V3‐V4 regions of the 16S ribosomal RNA (rRNA) gene in a prophylactic DSS‐induced colitis mouse model.

DSS administration led to significant dysbiosis, characterized by decreased operational taxonomic units (OTUs) and ACE index values compared to the control group. However, ECA@EcN treatment effectively preserved microbiota diversity and richness, suggesting its potential to counteract DSS‐induced microbial disturbances (Figure S23A,B, Supporting Information). Furthermore, nonmetric multidimensional scaling (NMDS) plots indicated distinct clustering patterns across treatment groups, with the ECA@EcN group closely aligning with the control group, highlighting its restorative effect on microbiota composition (Figure S23C, Supporting Information).

Further taxonomic analysis at the family and genus levels revealed notable shifts in bacterial populations following ECA@EcN treatment (Figure S23D,E, Supporting Information). The relative abundance of beneficial bacteria increased, while pathogenic bacteria levels decreased compared to the DSS group (Figure S23F–H, Supporting Information). These findings strongly suggest that ECA@EcN contributes to restoring microbial homeostasis, which is critical for mitigating colitis progression and maintaining gut health.

**Figure 7:**
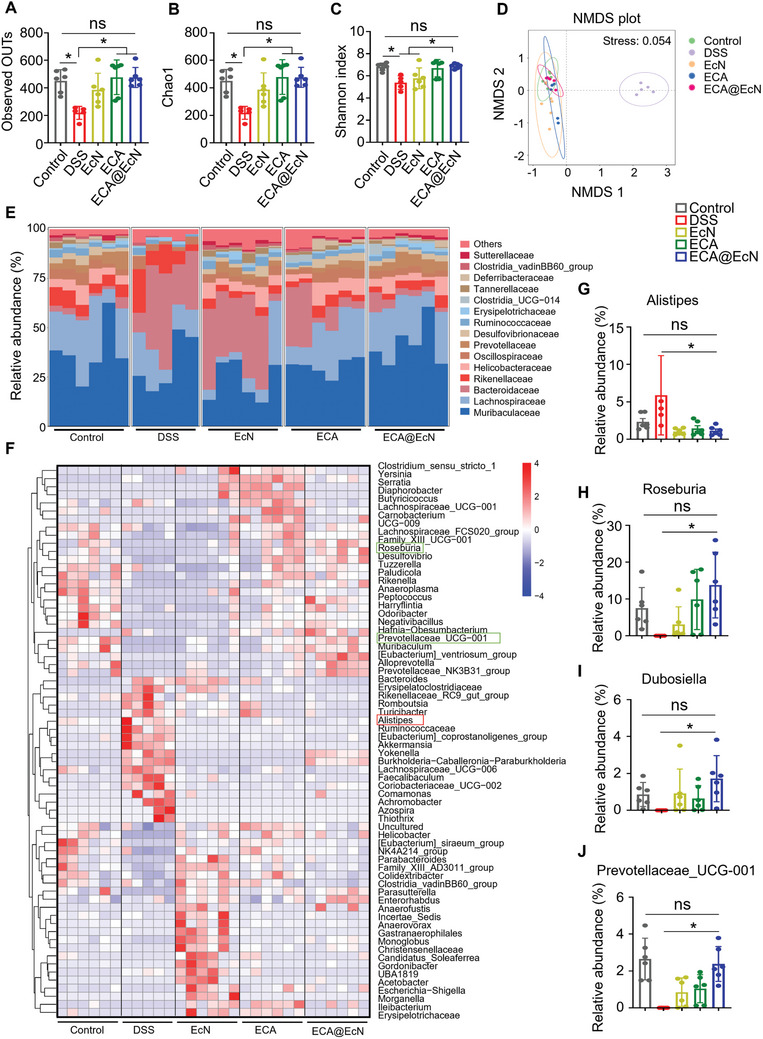
ECA@EcN modulates gut microbiota composition during IBD treatment. A–C) α‐diversity metrics displayed as observed operational taxonomic unit (OTU), Chao index, and Shannon index. D) NMDS (non‐metric multidimensional scaling) plot showing gut microbiome β‐diversity. E) Relative abundance of gut microbiota at the family level across treatment groups. F) Heatmap illustrating relative abundances of gut microbiota at the genus level. G–J) Relative abundances of selected taxa. Data are presented as the means ± SD (n = 5 or 6). Statistical analysis was performed using one‐way ANOVA. *p < 0.05; ns, not significant.

Building on these findings, the therapeutic potential of ECA@EcN was further investigated in a DSS‐induced colitis model. As shown in **Figure** **7**A–C, α‐diversity analysis revealed significant increases in OTU richness and the Chao1 and Shannon indices in the ECA@EcN‐treated group compared to the DSS group, indicating enhanced microbial diversity. While other treatment groups exhibited partial improvements, the microbiota composition of the ECA@EcN group most closely resembled that of the control group. These results highlight the superior capacity of ECA@EcN to restore gut microbial equilibrium. Additionally, β‐diversity analysis using NMDS plots demonstrated distinct gut microbiota composition clusters in the DSS‐treated groups. Notably, the bacterial community structure in the ECA@EcN group closely aligned with that of the control group, indicating effective restoration of gut microbiota (Figure 7D).

Detailed taxonomic analysis at the family (Figure 7E) and genus (Figure 7F) levels confirmed that DSS‐induced dysbiosis was significantly alleviated by ECA@EcN treatment. Specifically, the relative abundance of Alistipes, a genus linked to inflammatory responses and colorectal cancer,^[^ ^37^ ^]^ was markedly reduced in the ECA@EcN‐treated group (Figure 7G). Concurrently, the abundance of beneficial probiotic genera such as Roseburia (Figure 7H), Dubosiella (Figure 7I), and Prevotellaceae_UCG‐001 (Figure 7J) increased significantly, which are known to produce short‐chain fatty acids (SCFAs) and promote an anti‐inflammatory gut environment.^[^ ^38^ ^]^ These results further underscore the efficacy of ECA@EcN in re‐establishing a health‐supporting gut microbiota profile.

In summary, ECA@EcN treatment effectively enhances the relative abundance of beneficial bacteria, reduces pathogenic bacterial populations, and improves overall microbiota richness and diversity. These findings confirm the potential of ECA@EcN as a promising therapeutic strategy for restoring gut microbiota homeostasis and alleviating IBD symptoms. Future investigations focusing on long‐term effects and human clinical trials could further validate its translational potential.

## 3 Conclusion

In conclusion, we developed a novel polyphenol‐nanozyme‐armored probiotic (ECA@EcN) that demonstrates significant potential in treating IBD through its dual action of alleviating intestinal inflammation and restoring microbiota balance. This innovative platform leverages the binding of ECA to the EcN surface via phenolic‐mediated interfacial and electrostatic interactions, offering a sustainable and promising therapeutic approach. The polyphenol nanozymes components of ECA exhibited potent ROS‐scavenging activities,^[^ ^7^, ^39^ ^]^ effectively mitigating oxidative stress and thereby protecting both colonic epithelial cells and EcN from ROS‐induced damage. Mechanistically, ECA conferred protection to EcN by inhibiting the activation of the Flagellar Assembly and Branched‐Chain Amino Acid Synthesis pathways, crucial for bacterial resilience under oxidative stress.

Encapsulation with ECA significantly enhanced EcN's resilience to the harsh gastrointestinal environment by forming a protective barrier, while its improved mucoadhesive properties facilitated prolonged intestinal retention and precise targeting of inflamed colonic regions. These attributes collectively contributed to enhanced therapeutic efficacy in both prophylactic and therapeutic DSS‐induced colitis models, leading to significant alleviation of clinical symptoms, preservation of colon integrity, and reduction in inflammatory cytokine levels.

High‐throughput 16S rRNA gene sequencing revealed that ECA@EcN treatment led to a significant shift in gut microbiota composition, characterized by an increased abundance of beneficial bacteria, such as Roseburia and Dubosiella, and a reduced presence of pathogenic taxa, including Alistipes. This rebalancing of the gut microbiome is critical for restoring microbial homeostasis and, consequently, alleviating IBD symptoms and maintaining gut health.

Overall, ECA@EcN represents a promising strategy for enhancing the efficacy of probiotics in addressing intestinal inflammation and microbiota dysbiosis. The synergistic effects of polyphenol‐nanozyme components and probiotics provide a novel and effective approach to IBD management. Future studies should focus on optimizing dosage, assessing long‐term safety, and evaluating its clinical potential in human trials to translate these findings into practical therapeutic solutions.

## 4 Experimental Section

### Materials

The following chemical and biological reagents were used in this study: Epigallocatechin‐3‐gallate (EGCG) was obtained from Energy Chemical (Shanghai, China). Dextran sulfate sodium (DSS, MW: 36–50 kDa), MP Biochemicals. pepsin (Cat# P110927), recombinant Trypsin (Cat# R141084), Vanadium trichloride (Cat# V498277), Palladium nitrate, dihydrate (Cat# P105995), Ruthenium Trichloride Hydrate (Cat# R109234), Molybdenum(III) chloride (Cat# M498777), Rhenium(III) chloride (Cat# R486653), Cerium(III) chloride heptahydrate (Cat# C104760), Cobalt chloride hexahydrate (Cat# C116457), Rhodium trichloride (Cat# R132432), Iron sulfate heptahydrate (Cat# F116338), and Gold chloride trihydrate (Cat# G141105) were purchased from Aladdin Biochemical Technology Co., Ltd. (Shanghai, China). 2,2‐diphenyl‐1‐picrylhydrazyl (DPPH), 2,2'‐azinobis‐3‐ethylbenzothiazoline‐6‐sulfonic acid (ABTS, Cat# 30931‐67‐0), Potassium persulfate (K2S2O8, Cat# 7727‐21‐1), Bile salt (Cat# B875069), and Chitosan (CS, < 200 mPa.s) were purchased from Macklin Biochemical Co., Ltd. (Shanghai, China). Dimethyl sulfoxide (DMSO) and N,N‐dimethylformamide (DMF) were provided by Sigma‐Aldrich. Mouse IL‐6 (Cat# EK206), IL‐1β (Cat# EK201B), and TNF‐𝛼 (Cat# EK282) ELISA kits were purchased from MultiSciences (Lianke) Biotech Co., Ltd. The *Escherichia coli* Nissle 1917 (EcN) strain was purchased from Mingzhou Biological Technology Co., Ltd. (Ningbo, China). Propidium iodide (PI, Cat# 40711ES) was acquired from Yesen Biotechnology Co., Ltd. (Shanghai, China). 3,3′,5,5′‐Tetramethylbenzidine (TMB: ST746), DCFH‐DA fluorescent probe, Total SOD Assay Kit with WST‐8 (Cat# S0101S), Catalase Assay Kit (Cat# S0051), Hoechst 33342 (Cat# C1028), and DAPI fluorescent probe were acquired from Beyotime Biotechnology Co., Ltd. (Shanghai, China). MitoSOX reagent (Cat# M36008) was acquired from Thermo Fisher Scientific, Inc. (Boston, MA, USA). Cy5‐SE (16244‐5 mg) was purchased from CSN Pharm (Chicago, CA, USA). Commercial assay kits for ALT/GPT (Cat# C009‐2‐1), AST/GOT (Cat# C010‐2‐1), and T‐BIL (Cat# C019‐1‐1) were purchased from Nanjing Jiancheng (Nanjing, China). All other reagents were obtained from Beyotime Biotechnology Co., Ltd. (Shanghai, China), unless otherwise stated.

### Animals

Male C57BL/6 mice (6–8 weeks old, 20 ± 2 g) were purchased from Gempharmatech Co., Ltd. (Nanjing, China). The mice were acclimated in a specific pathogen‐free (SPF) facility for one week before the experiments. All animal procedures were conducted following the Institutional Animal Care and Use Committee (IACUC) guidelines of The First Affiliated Hospital of Anhui Medical University.

### Synthesis and Characterization of ECA

To prepare nanozymes, metal solution (1.13 mm) was slowly added to the citrate buffer, and the reaction mixture was stirred at 90 °C for 30 min.^[^ ^7^ ^]^

EGCG (200 mg) was oxidized via heating to generate EGCG quinone (QEGCG). CS (100 mg) was dissolved in 1.0% acetic acid (pH 4.0) and added dropwise to QEGCG in PBS (pH 7.4) under continuous stirring (1000 rpm) for 6 h to form EC. The EC‐to‐AuNP mass ratio was maintained at 1:0.57. EC and AuNPs were mixed in PBS (pH 7.4) under gentle stirring (1000 rpm) for 6 h. The ECA product was collected by centrifugation and washed with PBS to remove unbound materials.

The size, zeta potential, and morphology of ECA were analyzed using dynamic light scattering (DLS, NS‐90Z Plus, Omec, China) and transmission electron microscopy (TEM, JEM‐2100F, JEOL, Japan). The infrared spectrogram of ECA was characterized with an infrared spectrometer (Nicolet IS10, Thermo, USA). The XRD pattern was obtained by X‐ray diffractometry (D8 ADVANCE X, Bruker, Germany). Elemental analysis was obtained with an X‐ray photoelectron spectrometer (Kratos AXIS Ultra DLD, UK). The UV–vis absorption spectra of ECA were recorded using an ultraviolet spectrophotometer (MAPADA, P4 pc). The loading efficiency of EGCG was determined using a fluorescence quenching assay (excitation: 250 nm; emission: 300–450 nm).

### Preparation and Characterization of ECA@EcN

EcN cells were initially grown in LB medium under shaking conditions at 200 rpm and 37 °C until reaching an OD600 of 1.0‐1.2. After centrifugation at 4200 ×*g* for 5 min, the cells were washed twice with PBS and resuspended in PBS (pH 7.4). ECA solution (5 mM) was added, and the mixture was gently vortexed for 30 min at 37 °C to allow for ECA@EcN formation. The final product was centrifuged, washed with PBS, and characterized using TEM, DLS, and scanning electron microscopy (SEM, FEI Quanta 200F, NL). EcN cells were labeled with Hoechst 33342, and ECA with Cy5‐SE to confirm coating via confocal laser scanning microscopy (CLSM).

### Antioxidant Capacity, Enzymatic Activity, Cytokines Release, and Cytoprotective Effect of ECA

DPPH and ABTS assays were performed to assess the scavenging capacity of AuNPs, EC, and ECA.^[^ ^40^ ^]^ SOD‐ and CAT‐like activities were measured using commercial kits.^[^ ^41^ ^]^ POD‐like activity was assessed by monitoring TMB oxidation inhibition.^[^ ^42^ ^]^

RAW264.7 cells were treated with LPS (500 ng mL^−1^) and IFN‐γ (30 ng mL^−1^) for 12 h, followed by EC and ECA (0.1 µmol L^−1^) treatment for another 12 h. qRT‐PCR was used to quantify IL‐6, IL‐1β, and TNF‐α expression. Primer sequences for qRT‐PCR are listed in Table S1 (Supporting Information).

The CCK‐8 assay was employed to assess the protective effect of ECA against ROS‐induced damage and evaluate cytotoxicity in vitro. RAW264.7 cells were treated with different treatments, incubated with DCFH‐DA (10 µM), and fluorescence intensities were measured by flow cytometry. MitoSOX reagent was used to evaluate mitochondrial ROS. RAW264.7 and HT29 cells were cultured in a confocal dish and treated with EGCG and ECA (100 µM) for 24 h. Subsequently, the cells were treated with H_2_O_2_ for 1 h, and then stained with MitoSOX reagent to observe ROS using a Confocal laser scanning microscope (CLSM, Leica, STED).

### Hemolytic Toxicity Assay

The hemolytic toxicity assay was conducted in accordance with a previous report.^[^ ^43^ ^]^ Blood samples were collected from healthy mice. All animal studies adhered strictly to protocols approved by the Institutional Animal Care and Use Committee at The First Affiliated Hospital of Anhui Medical University. Red blood cells (RBCs) were collected by centrifugation at 3000 rpm for 5 min and washed three times with PBS. For the assay, 100 µL of PBS, EGCG (2.2 mM), ECA (2.2 mM), and 1% Triton X‐100 were each added to a 96‐well plate containing 100 µL of mRBCs, respectively. The plate was incubated at 37 °C for 30 min. Following incubation, the plate was centrifuged, and the absorbance of the supernatant was measured at 415 nm using a microplate reader. The hemolysis percentage was calculated using the following Equation (1):

(1) $$\begin{array}{ccc} & & \%\;\mathrm{hemolysis}=(Asamples\;-APBS)\;\\ & & /\;(ATriton\;X-100\;-\;APBS)\;\times \;100 \end{array}$$

### ECA@EcN Against ROS‐Induced Damage and Environmental Assaults

After encapsulation of EcN with the ECA layer, the probiotic was diluted and inoculated into a 96‐well plate to achieve an OD600 value of ≈0.15, either with or without 100 µM of H_2_O_2_. The plate was incubated at 37 °C with gentle shaking. The OD600 values were monitored at 1‐h intervals over a period of 12 h using a microplate reader. Uncoated EcN was used as the control.

### Resistance Assay

EcN were added to LB medium containing 100 µM H_2_O_2_, a toluene‐water interface, or an acidic solution at pH 4 for 30 min. After that, EcN was collected by centrifugation and washed three times with PBS. The cells were then incubated with Hoechst 33342 for 1 h at 37 °C in the dark, followed by incubation with propidium iodide for 20 min. Finally, The EcN cells were rinsed three times with PBS to prepare them for cell viability measurement.

To evaluate the resistance of EcN and ECA@EcN to various simulated gastrointestinal conditions, equal amounts of each were subjected to different environmental challenges. For assessing resistance to SGF, EcN and ECA@EcN were placed in SGF (pH 1.5) with 0.32% pepsin and incubated at 37 °C while shaking at 225 rpm. Samples of 50 µL were taken at specific time intervals, rinsed with PBS, and plated on LB agar in tenfold serial dilutions. Colonies were enumerated after 24 h of incubation at 37°C. To evaluate resistance to SIF and bile salts, EcN and ECA@EcN were incubated in SIF (pH 6.8) with trypsin (10 mg mL^−1^) and bile salts (0.4%) at 225 rpm and 37°C. At designated time points, the cultures were diluted and transferred to a 96‐well plate with an OD600 of ≈0.2. The plate was kept at 37 °C and gently shaken, with OD600 measurements taken every 30 min over a 2‐h period using a microplate reader. The stability of ECA was assessed by incubating it with PBS, SGF, bile salts, and SIF for 24 h. The particle size of ECA in these media was measured to detect any changes, thereby ensuring the consistency and integrity of the ECA formulation under different conditions.

### Mucoadhesive Evaluation In Vivo

To evaluate the ability of ECA@EcN to target inflamed site in the colon and assess its biodistribution, EcN cells were initially labeled with Cy5. Briefly, EcN (1 × 10^8^ CFU) were washed three times with PBS and then incubated with 0.13 µmol of Cy5‐SE for 1 h with gentle stirring to label the bacteria. Next, the Cy5‐labeled bacteria were washed with PBS three times to remove excess dye. The labeled EcN cells were orally administered, and at 4, 12, 24, and 48 h post‐administration, the distribution of EcN was monitored using In Vivo Imaging System (IVIS). Furthermore, after 48 h, the mice were euthanized, and the organs, including the heart, kidneys, lungs, spleen, liver, and gastrointestinal tract were harvested. The fluorescence intensities in each organ were isolated and imaged to determine the biodistribution of EcN and ECA@EcN. This method allows for a detailed evaluation of the targeting and retention capabilities of ECA@EcN within the inflamed colon, as well as its overall distribution throughout the body.

### Prophylactic and Therapeutic Efficacy of ECA@EcN Against DSS‐Induced Colitis

The prophylactic and therapeutic efficacy of ECA@EcN against DSS‐induced colitis was evaluated using a well‐established murine model. Mice were randomly assigned to five experimental groups: Control, DSS, EcN, ECA, and ECA@EcN. In the prophylactic regimen, EcN and ECA formulations (bacterial dose: 1 × 10^8^ CFU; ECA: 50 µM kg^−1^) were administered via oral gavage every two days throughout the study. A freshly prepared 3% DSS solution was provided in the drinking water for one week to induce colitis, except for the Control group, which received plain water. After the DSS treatment phase, normal drinking water was provided to all groups. To eliminate the influence of oral gavage, a second prophylactic model was developed by incorporating EcN, ECA, or ECA@EcN into drinking water. In brief, EcN, ECA, and ECA@EcN were introduced into the drinking water at concentrations of 2 × 10⁷ CFU mL^−1^ (EcN) and 0.2 mM (ECA suspended in a 0.9% normal saline solution), respectively.

For therapeutic assessment, the same DSS‐induced colitis model and grouping were employed. After the DSS exposure period, normal drinking water was provided, and the respective formulations were administered for five consecutive days. Throughout both studies, mice were monitored for changes in body weight, fecal consistency, and the presence of blood in the stool. Disease severity was quantified using the Disease Activity Index (DAI) based on established protocols.^[^ ^42^ ^]^ On the final day of each study, mice were euthanized to collect colonic tissues for length measurement. Additionally, blood, fecal samples from the colon, and major organs (heart, liver, spleen, lungs, and kidneys) were harvested for further analysis.

### Histopathology Studies

The distal colons and vital organs were fixed in 4% paraformaldehyde, embedded in paraffin, and sectioned for hematoxylin and eosin (H&E) staining. The stained tissues were examined under an optical microscope, and histopathological analysis of colon damage was performed according to established methods.^[^ ^15^ ^]^

### Immunofluorescence

Immunofluorescence imaging was employed to detect the expression of CD68 (Cell Signaling Technology, #97778T, 1:200), F4/80 (Cell Signaling Technology, #70076T, 1:200), ZO‐1 (Cat# GB111402, Servicebio, 1:200), and occludin‐1 (Cat# 13050‐1‐AP, Proteintech, 1:200) in colon tissue by using primary antibodies. The assay was performed according to the manufacturer's instructions (AiFang Biological, AFIHC024).

### Enzyme‐Linked Immunosorbent Assay (ELISA)

To assess cytokine levels in serum samples, whole blood was centrifuged at 3000 g for 20 min at 4 °C. Serum concentrations of IL‐6, IL‐1β, and TNF‐α were quantified using ELISA kits according to the manufacturer's instructions.

### Transcriptome Sequencing for Receiving CA@EcN

EcN cells were harvested from Luria‐Bertani (LB) agar plates and cultured in LB medium with shaking at 200 rpm at 37 °C until the optical density (OD) at 600 nm reached 1.0–1.2, indicating the stationary growth phase. The bacterial culture was centrifuged at 4200 ×g for 5 min, washed twice with PBS, and resuspended in PBS at pH 7.4 to serve as the control group. For test samples, a 5 mM ECA solution in PBS was added to the EcN suspension, followed by gentle vortexing at 37 °C for 30 min to facilitate the formation of ECA@EcN complexes. The resulting suspension was centrifuged, washed three times with PBS to remove unbound ECA, and stored for further analysis.

Both untreated EcN and ECA@EcN samples were subsequently exposed to 100 µM H₂O₂ in PBS under vigorous agitation at 37 °C for 30 min to simulate oxidative stress conditions. After treatment, the samples were centrifuged, washed twice with PBS, and rapidly frozen in liquid nitrogen to preserve RNA integrity. Total RNA was extracted using the RNAprep Pure Cell/Bacteria Kit (TIANGEN, Beijing, China; Cat. No. DP430) according to the manufacturer's instructions. RNA purity and quantity were assessed with a NanoDrop 2000 spectrophotometer (Thermo Scientific, USA), while RNA integrity was evaluated using an Agilent 2100 Bioanalyzer (Agilent Technologies, USA). Samples meeting the quality criteria were selected for library construction, during which ribosomal RNA was removed using the TIANSeq rRNA Depletion Kit (TIANGEN, Beijing, China).

Library preparation was conducted using the VAHTS Universal V6 RNA‐seq Library Prep Kit following the manufacturer's guidelines. The prepared libraries were subjected to transcriptome sequencing and subsequent bioinformatics analysis performed by OE Biotech Co., Ltd. (Shanghai, China).

### Microbiome Analysis

After various treatments, fecal samples were promptly frozen in liquid nitrogen and sent to Hangzhou Cosmos Wisdom Biotech Co and Shanghai OE Biotech Co for further processing. Sequencing libraries for the V3 and V4 regions were prepared using the Illumina NovaSeq platform (Illumina, San Diego, CA, USA). The 16S rRNA amplicons were generated with V3‐V4 region primers “ACTCCTACGGGAGGCAGCA” (forward) and “GGACTACHVGGGTWTCTAAT” (reverse). For bioinformatics analysis, raw FASTQ files underwent demultiplexing, quality filtering with Trimmomatic (version 0.33), and removal of contigs and chimeras using Cutadapt. QIIME2 was used to select a representative sequence from each OTU, which was then aligned with the Silva or Greengenes 16S rRNA database, applying a confidence threshold of 0.7. Community diversity was analyzed using indices such as ACE, Chao1, Simpson, Shannon, and Observed‐species. Ordination analysis, including UniFrac‐based Principal Component Analysis (PCA) and Nonmetric Multidimensional Scaling (NMDS), was conducted to visualize sample clustering.

### Statistical Analysis

Statistical analyses were performed using Student's t‐tests or one‐way ANOVA in GraphPad Prism (Version 9). Statistical significance was defined as *p* < 0.05. Data are presented as mean ±SD, and ns was defined as no significant difference.

## Conflict of Interest

The authors declare no conflict of interest.

## Author Contributions

D.X., P.W., and B.S. performed conceptualization. Y.Z., Z.Q., J.B., L.W., J.C., Z.Z., Q.W., W.S., Z.G., D.X., P.W., and B.S. performed methodology. Y.Z., Z.Q., J.B., L.W., Z.G., D.X., and P.W. performed investigation. Y.Z., Z.Q., J.B., L.W., J.C., Z.Z., Q.W., W.S., Z.G., D.X., P.W., and B.S. managed resources. Y.Z., J.B., L.W., D.X., P.W., and B.S. performed formal analysis. D.X., P.W., and B.S. performed visualization. D.X., P.W., and B.S. performed funding acquisition. Y.Z., Z.Q., J.B., D.X., P.W., and B.S. wrote‐original draft. Y.Z., Z.Q., J.B., D.X., P.W., and B.S. wrote, reviewed, and edited. D.X., P.W., and B.S. performed supervision. All the authors reviewed, edited, and approved the paper.

## Supporting information



Supporting Information

## Data Availability

The data that support the findings of this study are available from the corresponding author upon reasonable request.
