# Supplementary material for: Orally Administered Functional Polyphenol‐Nanozyme‐Armored Probiotics for Enhanced Amelioration of Intestinal Inflammation and Microbiota Dysbiosis
Source: Adv Sci (Weinh). 2025 Mar 11;12(17):2411939. doi: 10.1002/advs.202411939 (PMC12061243; doi:10.1002/advs.202411939)
Supplement: Supplementary file 1 — Supporting Information [file ADVS-12-2411939-s001.docx]

**Supplementary Materials**

**Orally Administered** **Functional Polyphenol-Nanozyme-Armored Probiotics for Enhanced Amelioration of Intestinal Inflammation and Microbiota Dysbiosis**

Yong Zhu^1,2,3^, Ziqu Fang^1,2,3^, Jie Bai^2,3^, Longhui Wang^1,2,3^, Jiaqing Chen^1,2,3^, Zehua Zhang^1,2,3^, Qiang Wang^1,2,3^, Weiwei Sheng^4^, Xueyin Pan^1,2,3^, Zhenyuan Gao^4^, Dengqiu Xu^1,2,3*^, Pengkai Wu^1,2,3*^, Beicheng Sun^1,2,3*^

^1^ Department of Hepatobiliary Surgery, The First Affiliated Hospital of Anhui Medical University, Hefei, Anhui 230022, China.

^2^ MOE Innovation Center for Basic Research in Tumor Immunotherapy, Hefei, Anhui 230022, China.

^3^Anhui Province Key Laboratory of Tumor Immune Microenvironment and Immunotherapy, Hefei, Anhui 230022, China.

^4^ Department of General Surgery, Department of General Surgery, The First Affiliated Hospital of Anhui Medical University, Hefei 230022, China

*Correspondence:

Dengqiu Xu, Ph.D.

The First Affiliated Hospital of Anhui Medical University

218 Jixi Road, Shushan, Hefei, China 230022

xudengqiu@nju.edu.cn

Pengkai Wu, Ph.D.

The First Affiliated Hospital of Anhui Medical University

218 Jixi Road, Shushan, Hefei, China 230022

wupengkai@ahmu.edu.cn

Beicheng Sun, Ph.D.

The First Affiliated Hospital of Anhui Medical University

218 Jixi Road, Shushan, Hefei, China 230022

sunbc@ahmu.edu.cn


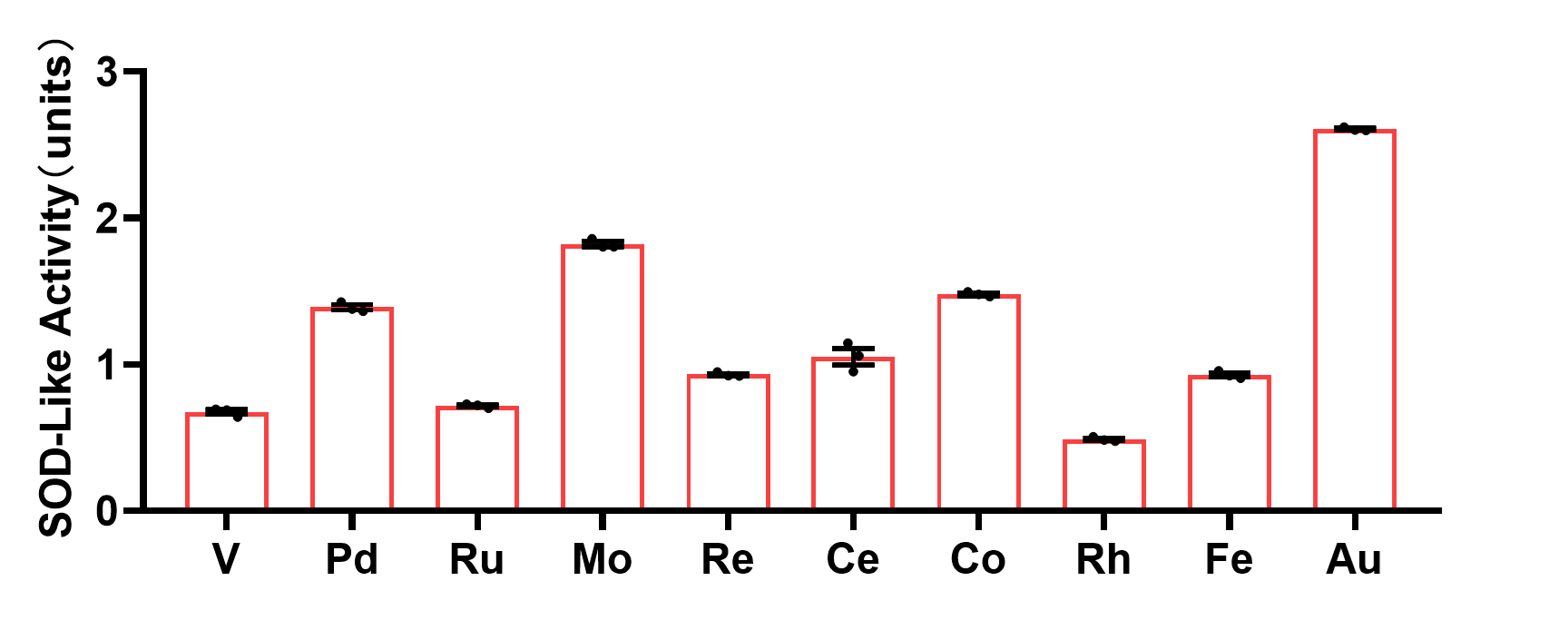


**Figure S1.** The superoxide dismutase (SOD)-like activities of nanozymes (n = 3).


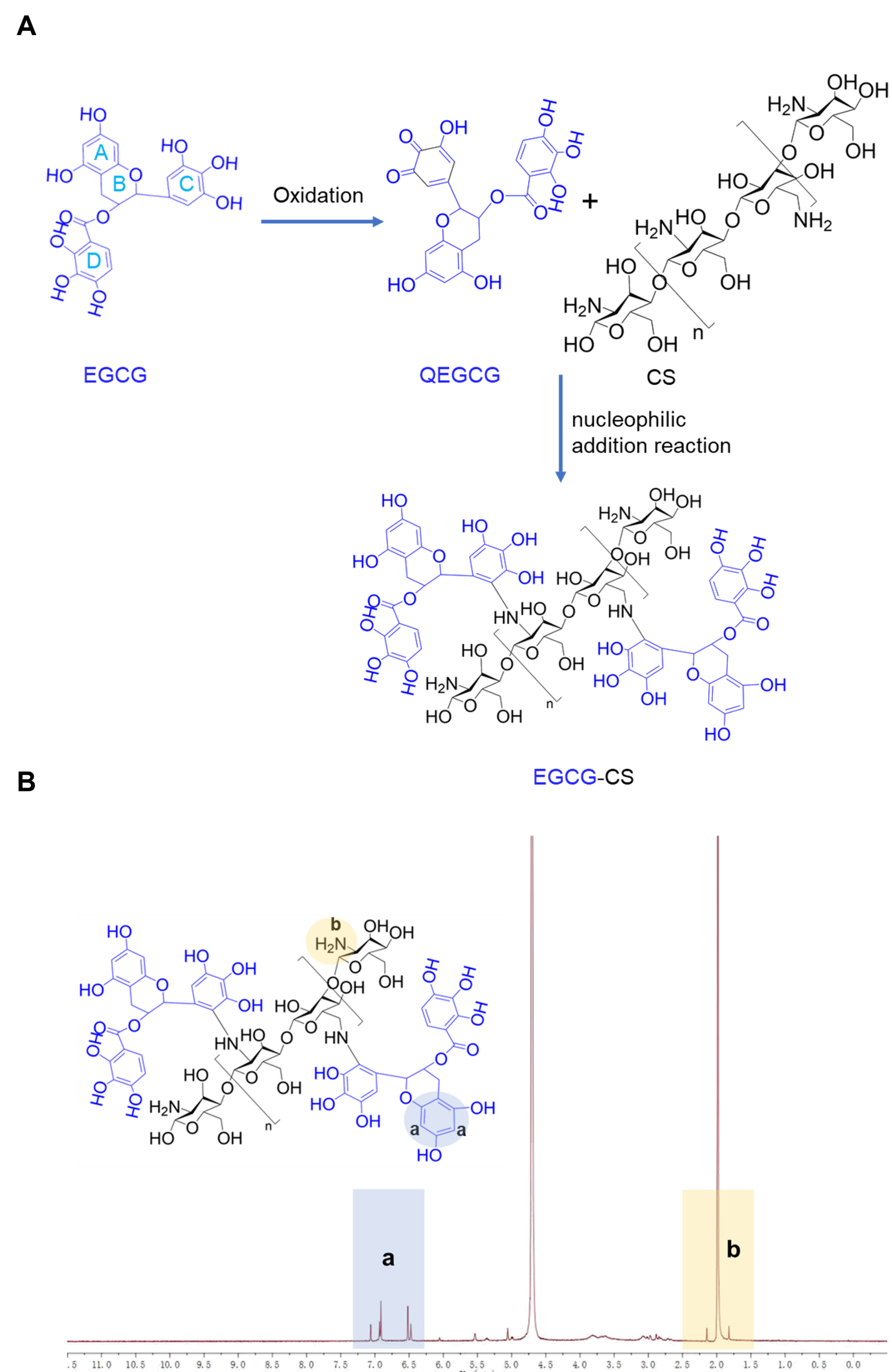


**Figure S2.** (A) Schematic illustrating the preparation process of EGCG-CS. The pyrogallol moiety (C ring) of EGCG was oxidized to form an ortho-quinone at the C ring (QEGCG) under pH 7.4. CS was specifically conjugated to the C ring of EGCG via a nucleophilic addition reaction. (B) The ^1^H NMR spectrum of EC.


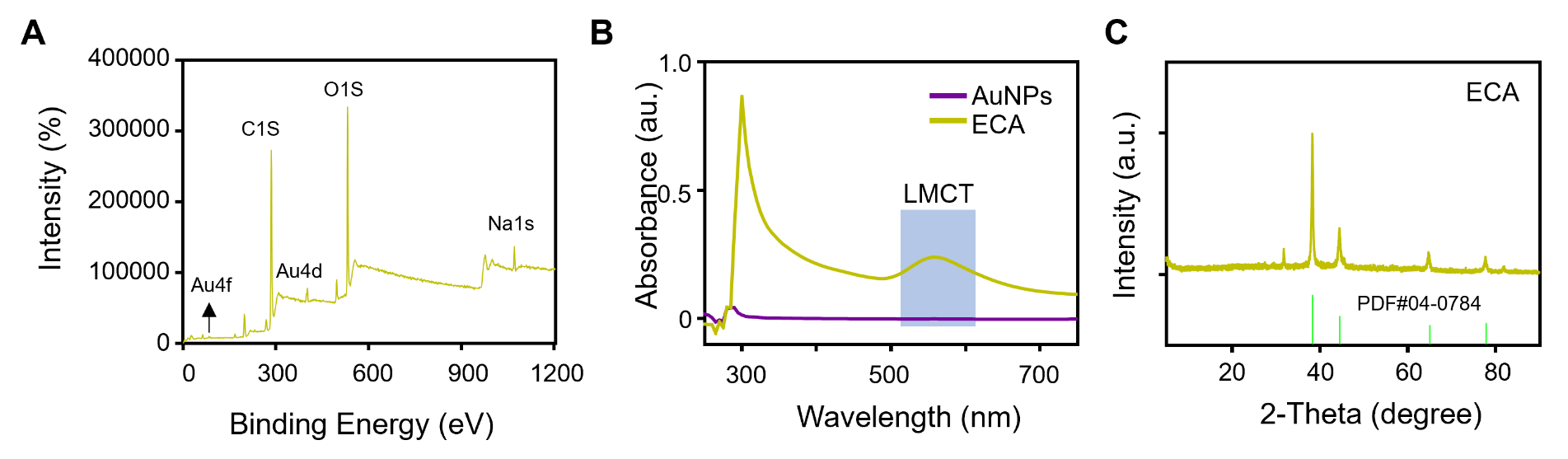


**Figure S3.** Characterization of ECA. (A) High-resolution XPS spectra of ECA. (B) UV-vis absorption spectra of AuNPs and ECA. (C) X-ray diffraction (XRD) patterns of ECA.


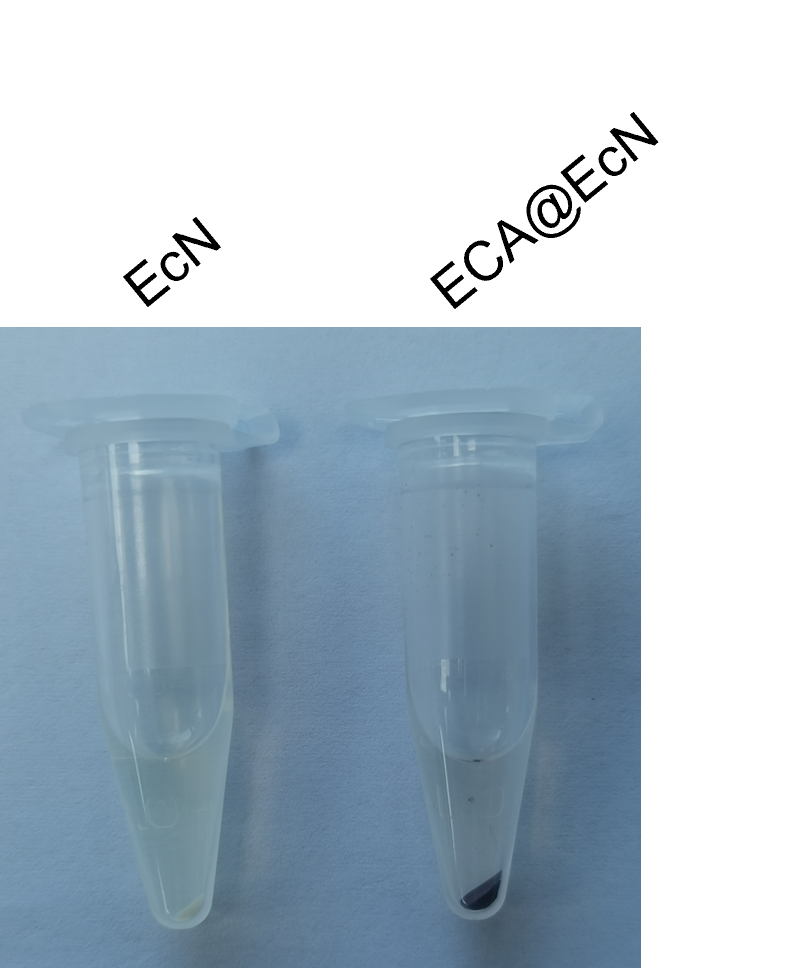


**Figure S4.** Suspension and deposition characteristics of native EcN and ECA@EcN. The suspensions of EcN turned black after encapsulation with ECA.


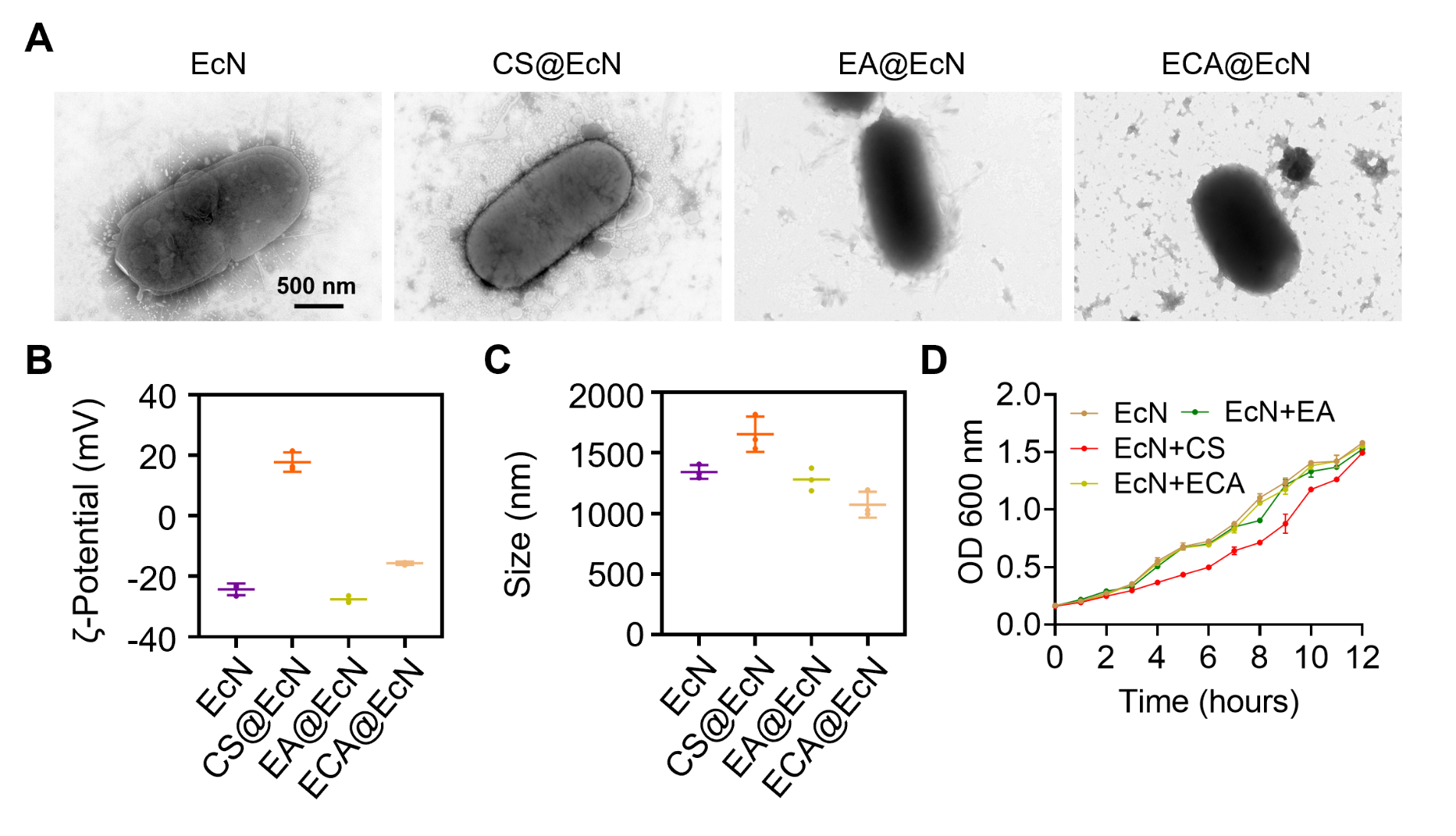


**Figure S5.** Characterization of coated EcN. (A) TEM images of EcN, CS@EcN, EA@EcN, and ECA@EcN. (B, C) Sizes and zeta potentials of EcN, CS@EcN, EA@EcN, and ECA@EcN. (D) Growth curves of EcN , CS@EcN, EA@EcN, and ECA@EcN at 37℃ (n =3).


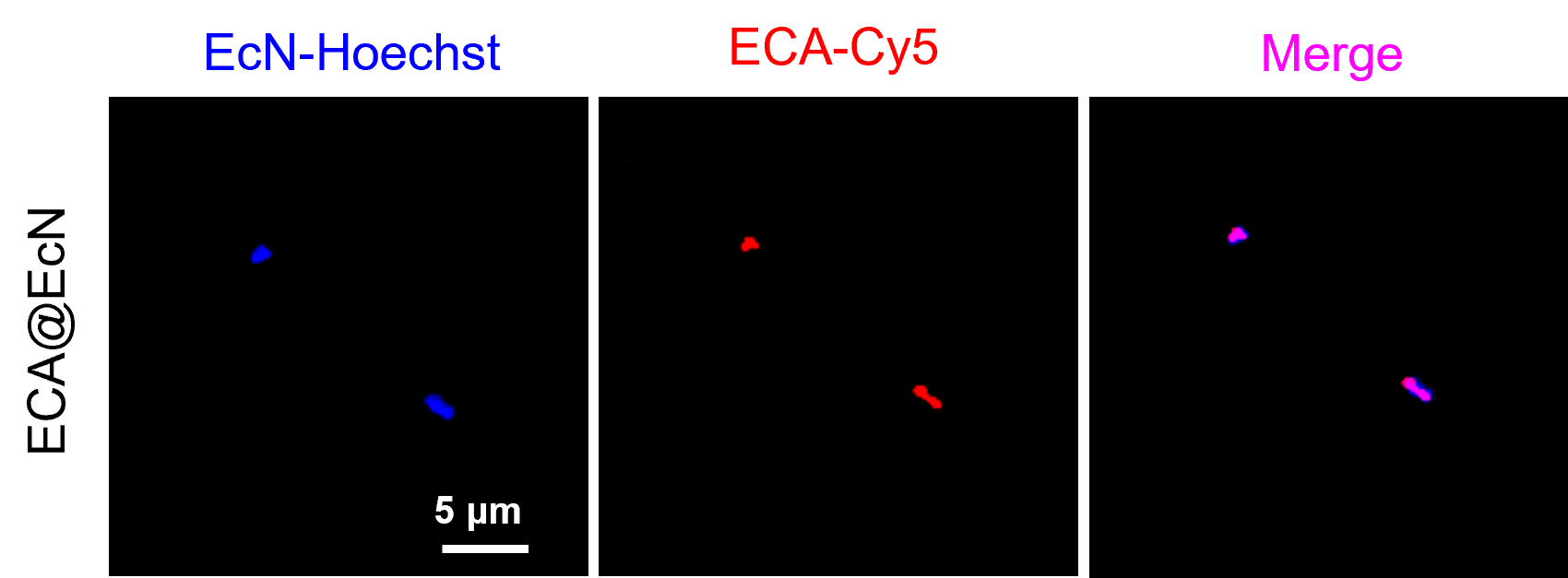


**Figure S6.** LSCM images of ECA@EcN. The blue fluorescence represents EcN cells stained with Hoechst 33342, while the red fluorescence represents the ECA layer stained with Cy5.


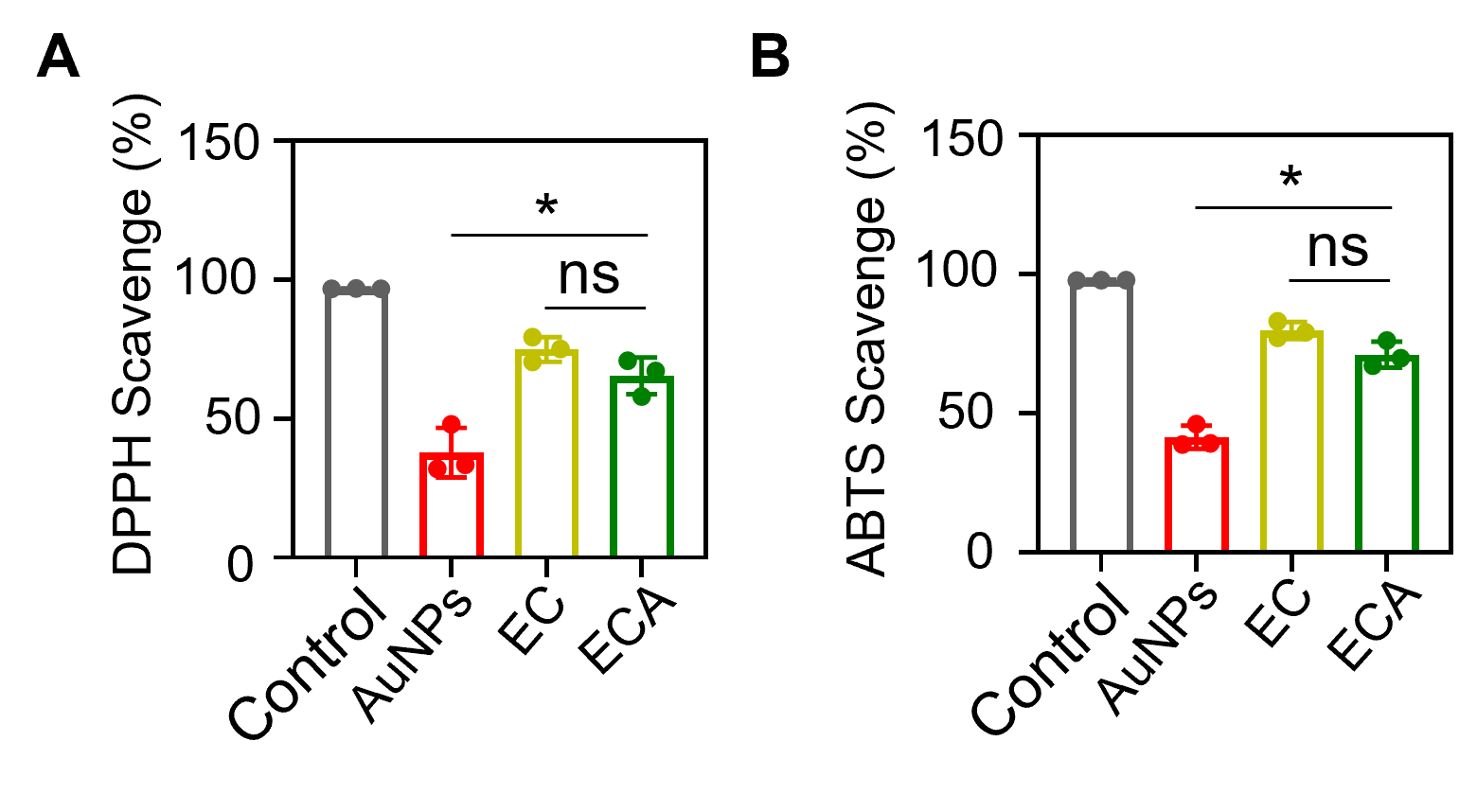


**Figure S7.** DPPH (A) radical and ABTS (B) radical scavenging activities of ECA. Data are presented as means ± SD. (n = 3). Statistical analysis was conducted using one-way ANOVA. *P < 0.05; ns, not significant.


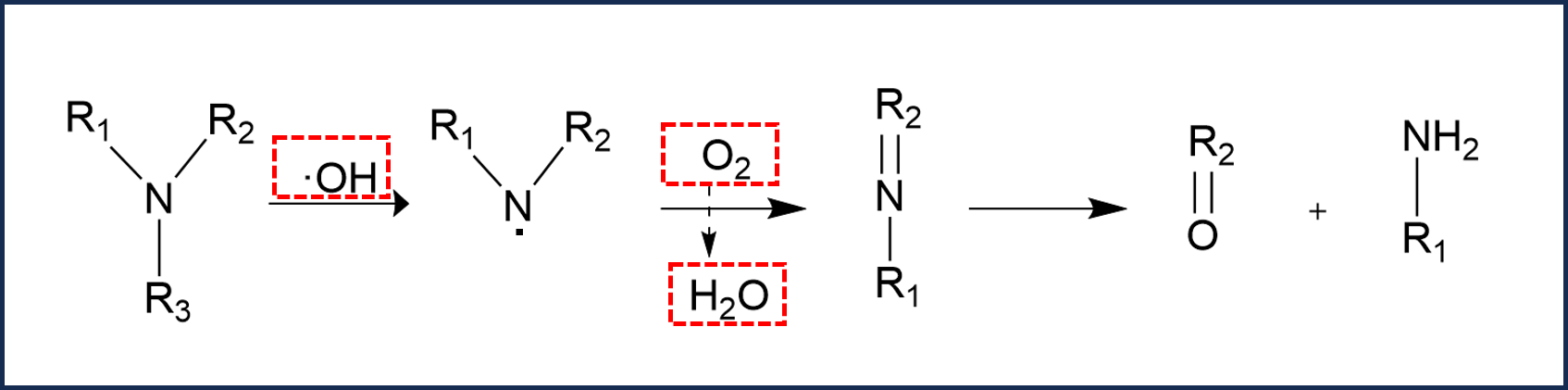


**Figure S8.** Mechanism illustrating AuNPs combined with CS transform peroxidase (POD)-like activity into catalase (CAT)-like activity.


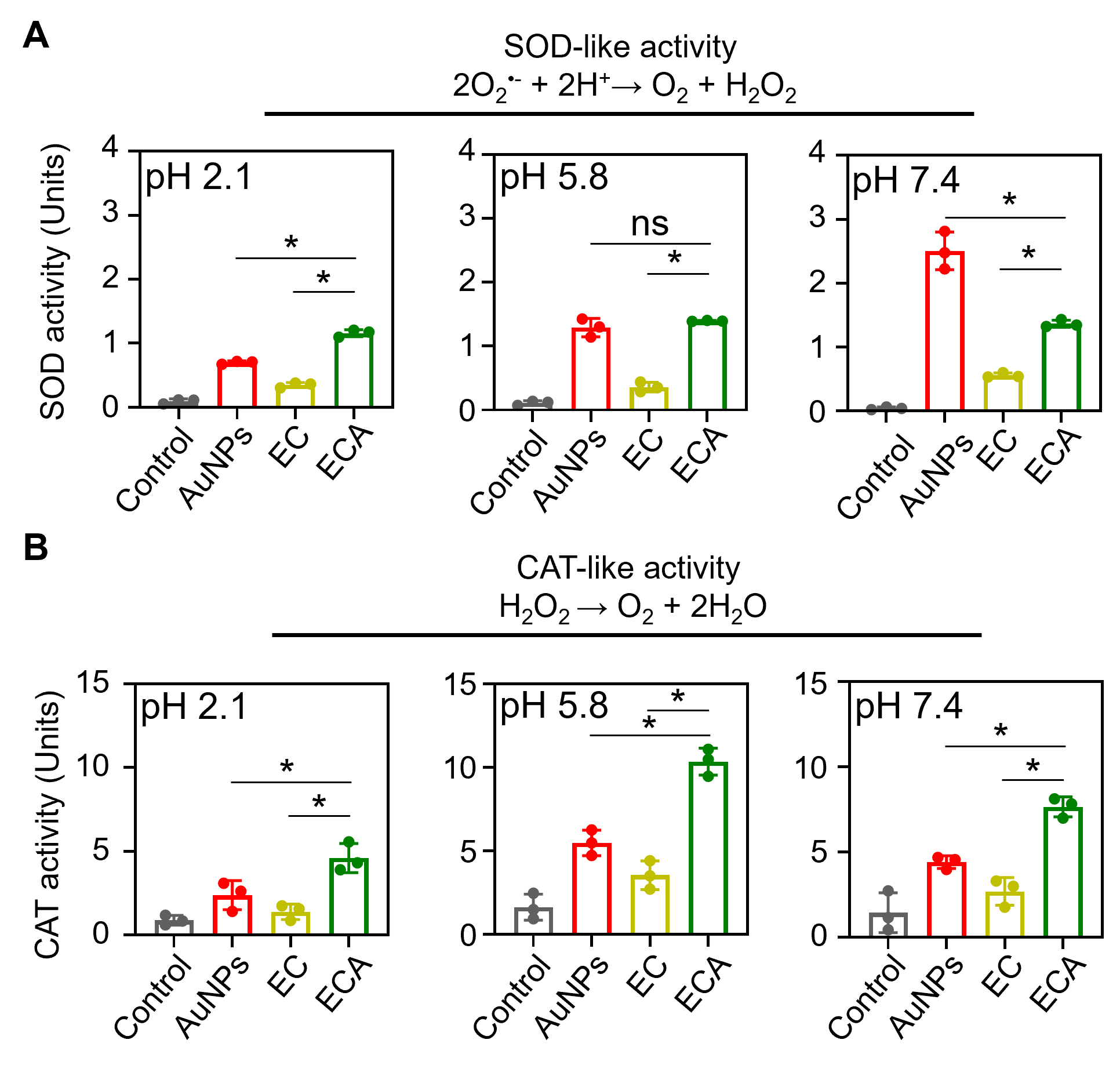


**Figure S9.** (A) The SOD-like activity and (B) the CAT-like activity of AuNPs, EC, and ECA under pH conditions of 2.1, 5.8, and 7.4. Data are presented as the means ± SD (n = 3). Statistical analysis was conducted using one-way ANOVA. *P < 0.05; ns, not significant.


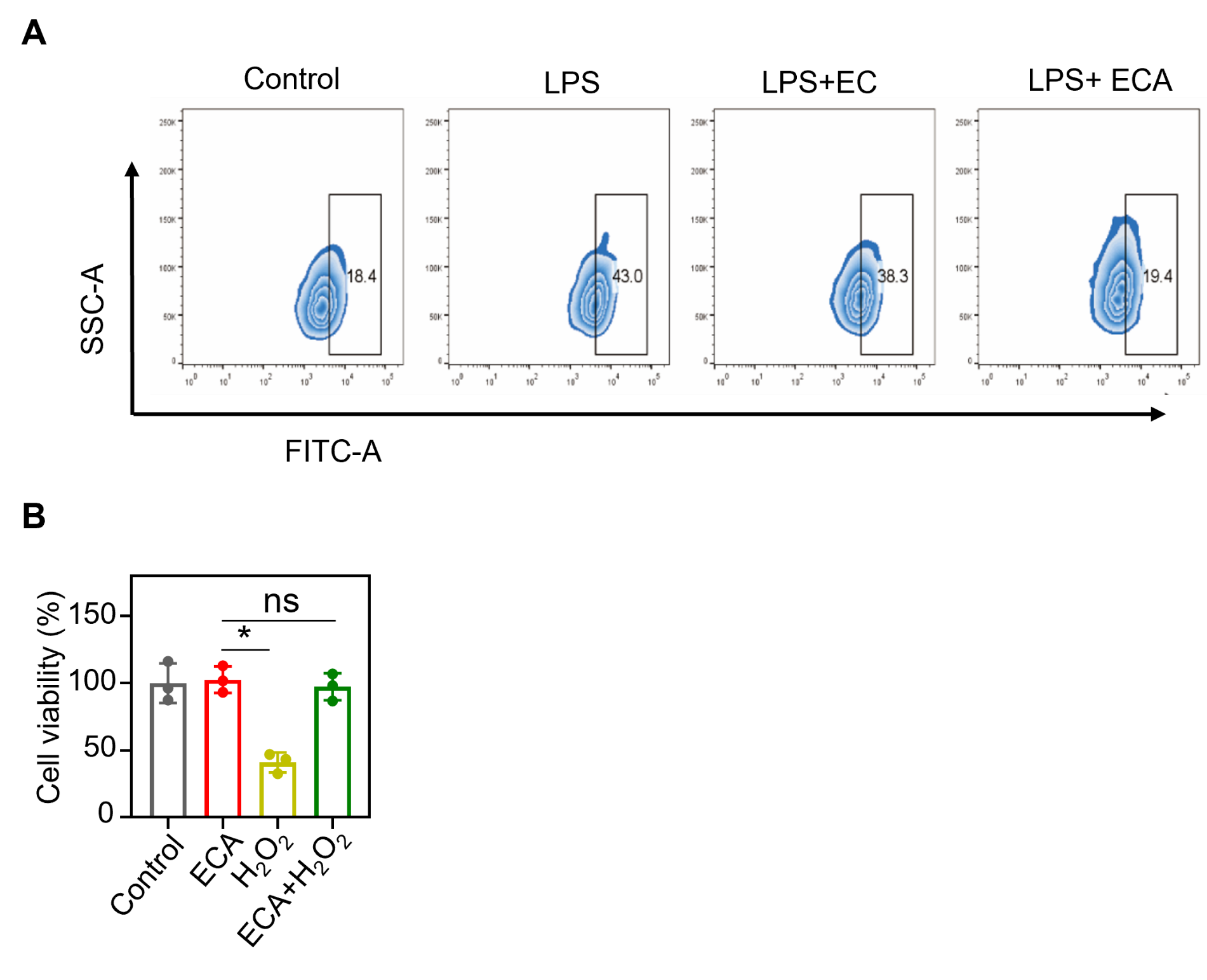


**Figure S10.** Antioxidant activities of ECA in vitro. (A) ROS production in RAW 264.7 cells under various treatment conditions were performed using flow cytometry. (B) Viability of SW480 cells following ECA treatment with or without H_2_O_2_ exposure. Data are presented as the means ± SD (n = 3). Statistical analysis was conducted using one-way ANOVA. *P < 0.05; ns, not significant.


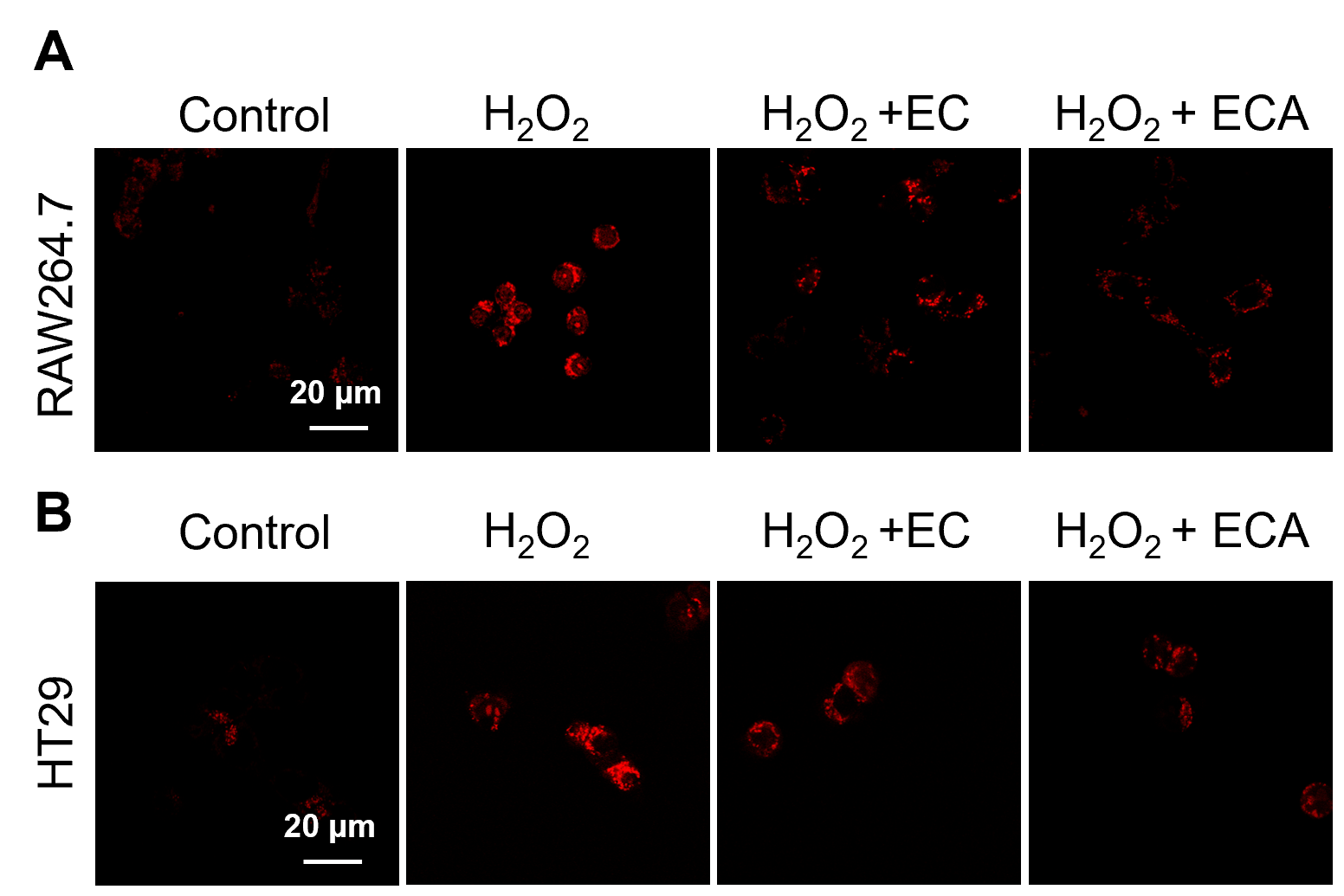


**Figure S11.** LSCM images of intracellular mitochondria ROS levels detected by MitoSOX Red dye in RAW 264.7 (A) and HT29 (B) cells.

**
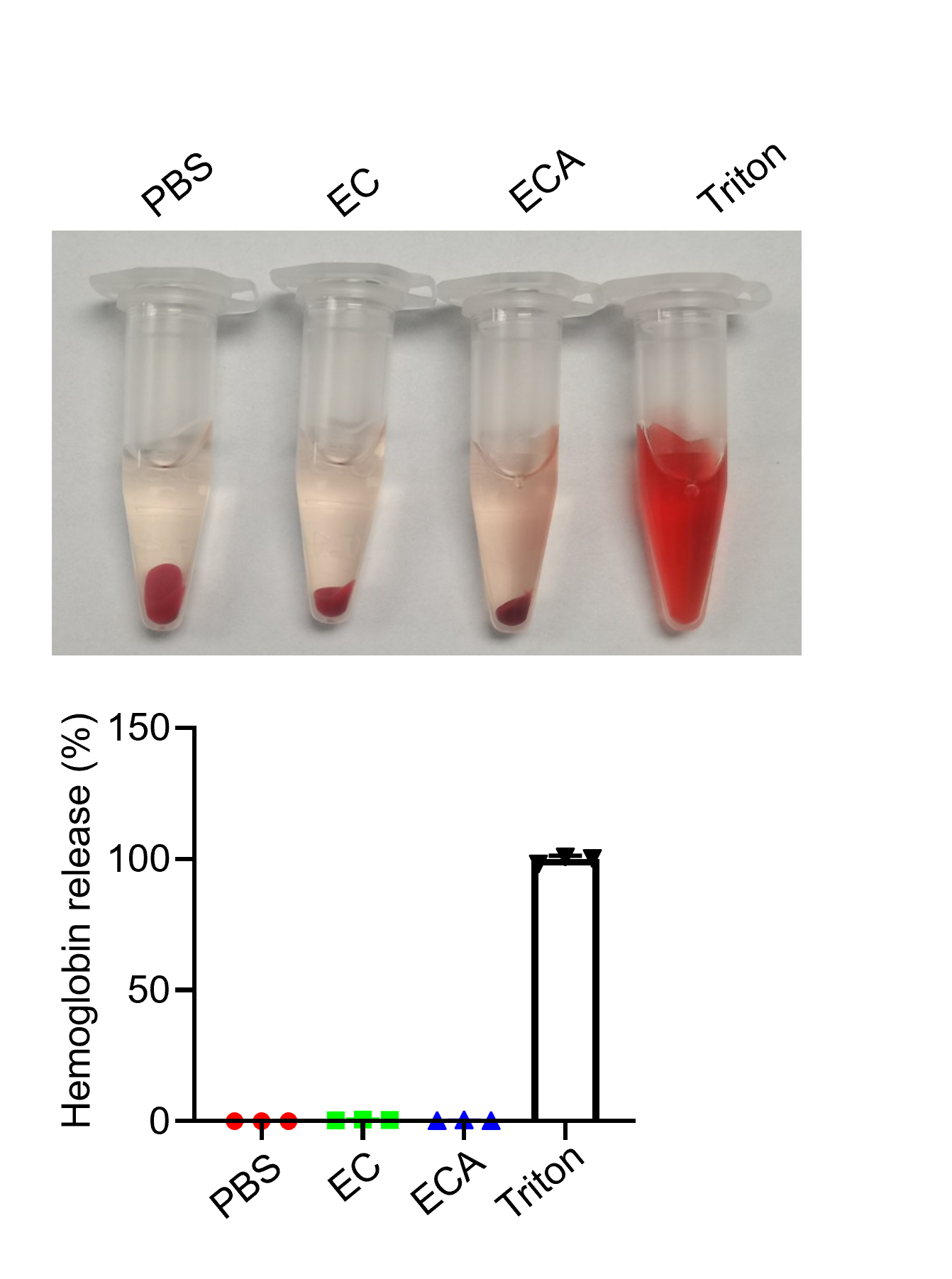
**

**Figure S12.** The hemolytic activity of EGCG and ECA on erythrocytes (n = 3). Data are presented as the means ± SD. Error bars represent standard deviations from three independent measurements.


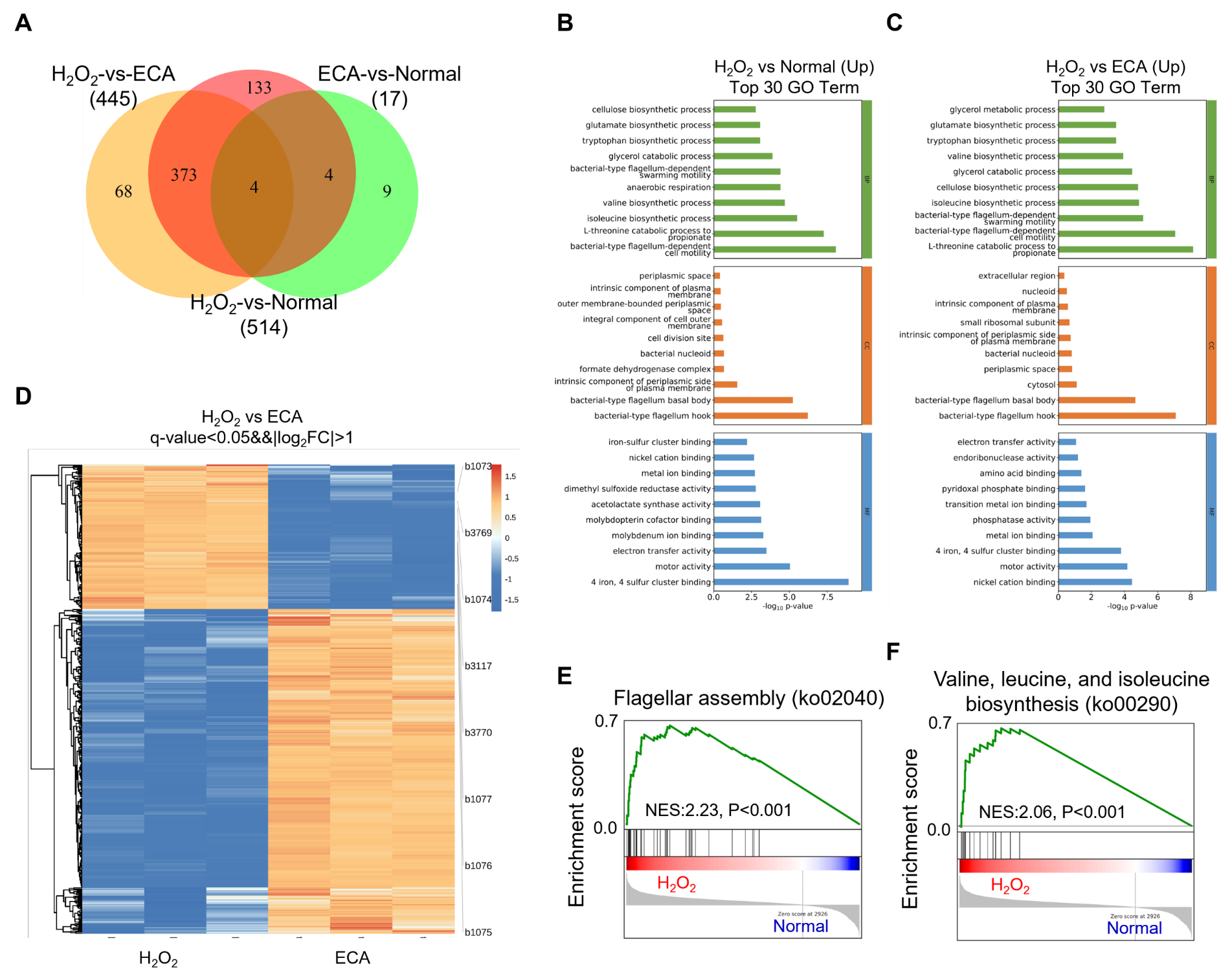


**Figure S13.** RNA sequencing (RNA-seq) analysis the protective mechanisms of ECA in EcN after H_2_O_2_ treatment. (A) Venn diagram from RNA-seq analysis showing significantly regulated genes. (B, C) GO term classifications of upregulated genes. (D) Heatmap depicting differentially expressed genes. (E) GSEA analysis of Flagellar assembly gene responses in H_2_O_2_-treated versus normal conditions. (F) GSEA analysis of Valine, Leucine, and Isoleucine Biosynthesis gene responses in H_2_O_2_-treated versus normal conditions.


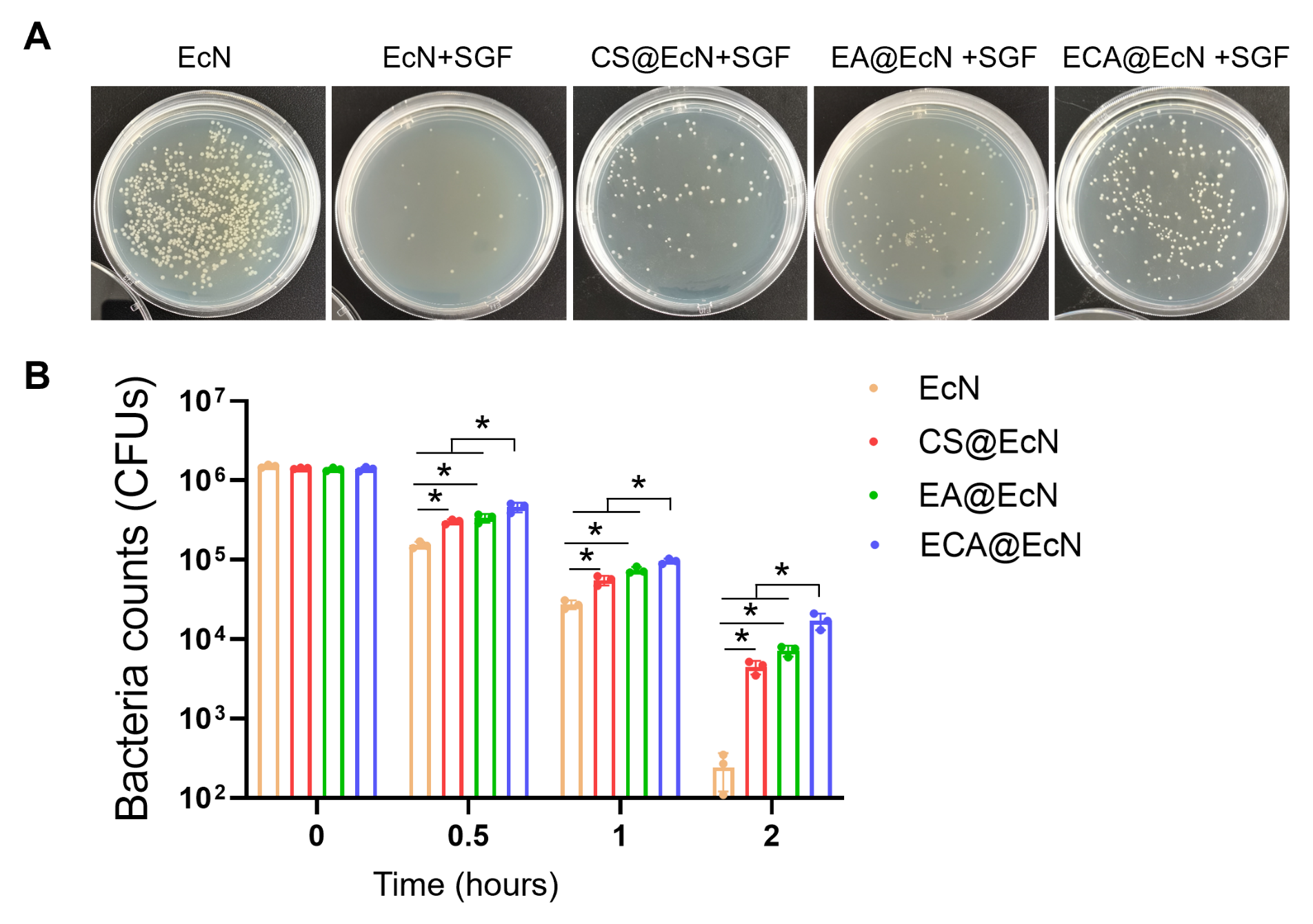


**Figure S14.** Resistance of coated EcN against simulated gastric fluid (SGF) challenges in vitro. (A) Photographs of bacterial colonies on agar plates showing EcN, CS@EcN, EA@EcN, and ECA@EcN after 0.5 h of incubation in SGF (pH 1.5) supplemented with pepsin. (B) Quantification of EcN, CS@EcN, EA@EcN, and ECA@EcN after SGF incubation (n = 3). Data are presented as the means ± SD. Statistical analysis was conducted using one-way ANOVA. *P < 0.05.


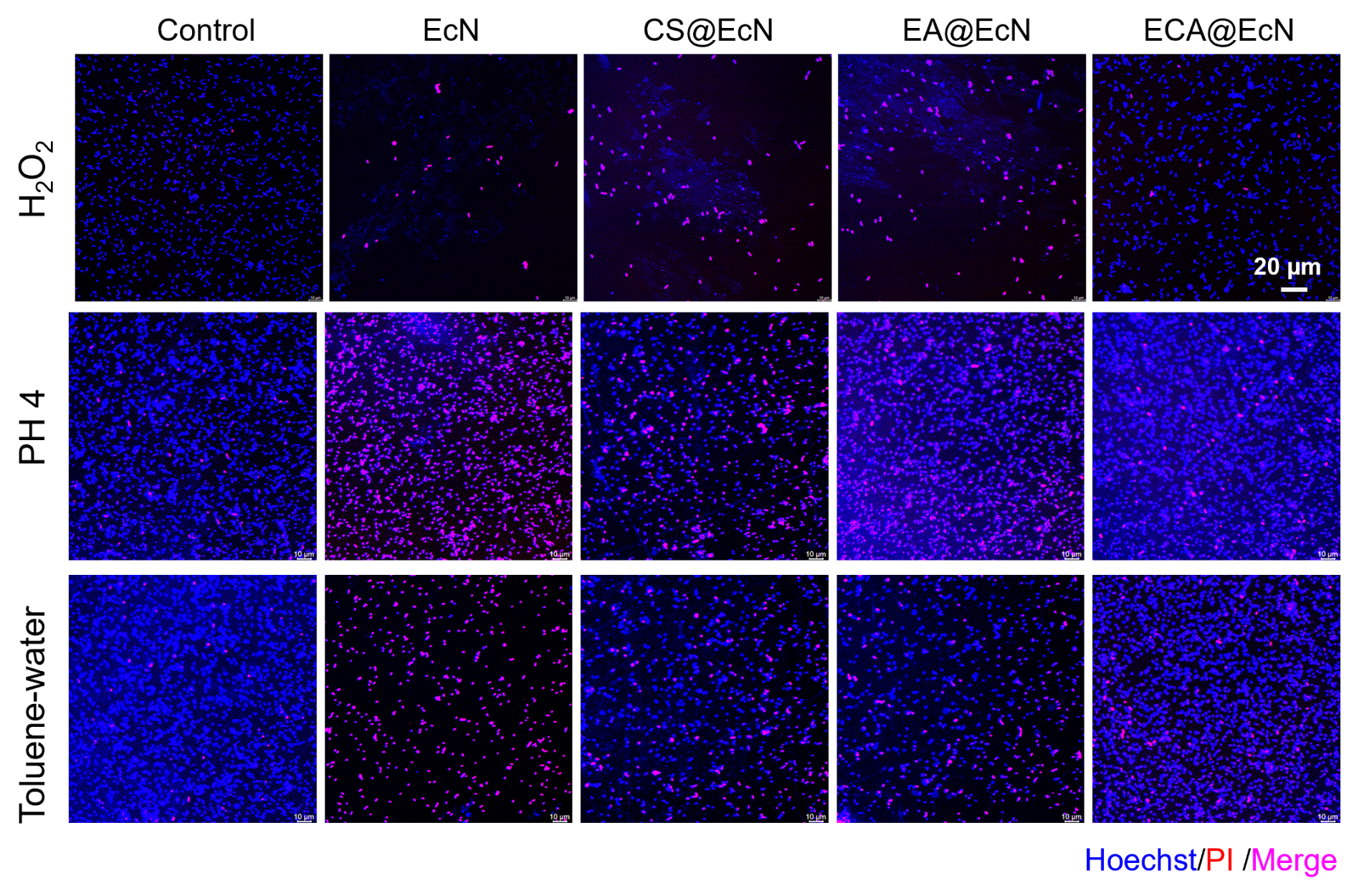


**Figure S15.** Representative confocal images of native EcN, CS@EcN, EA@EcN, and ECA@EcN stained with hoechst33342 (blue) and PI (red) after culturing in the LB medium containing H_2_O_2_ (100 μ M), a toluene-water interface, and an acidic solution at pH 4. Scale bar: 20 μm.


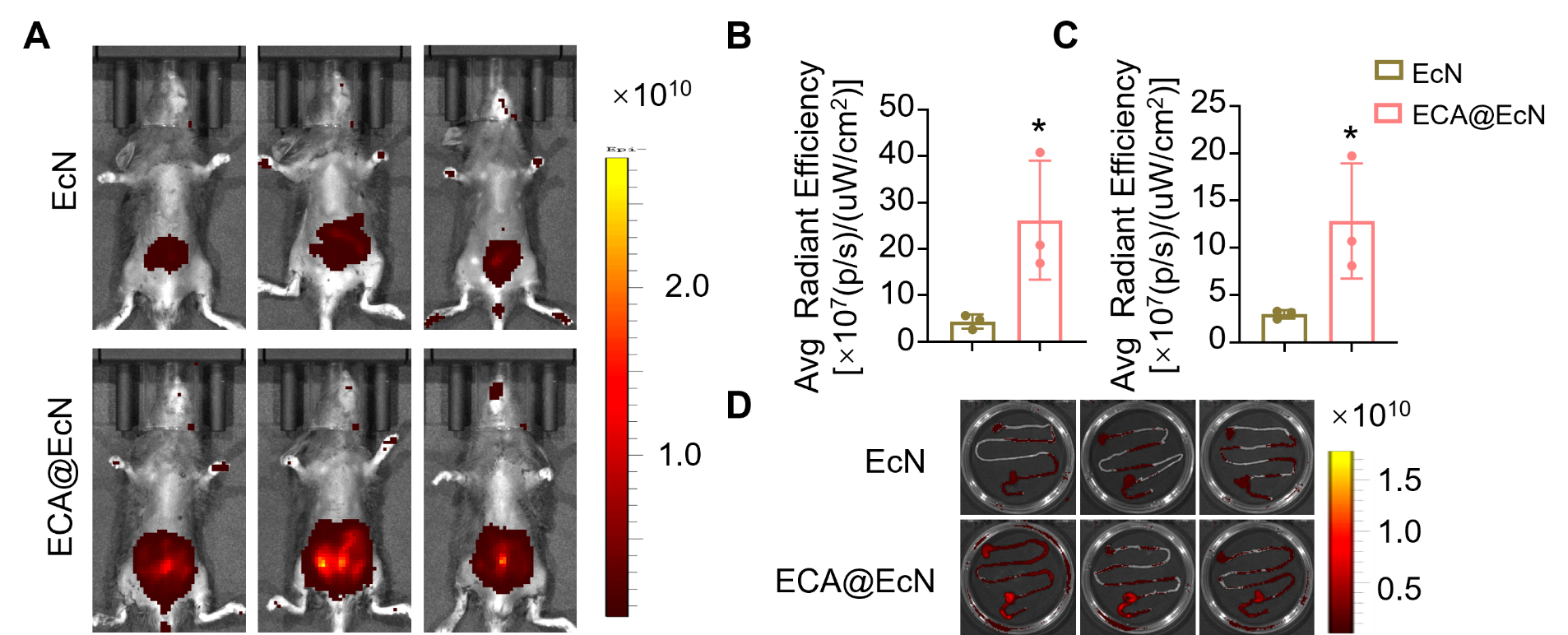


**Figure S16.** Biodistribution of ECA@EcN is depicted. (A, B) Chemiluminescence images and region-of-interest (ROI) analysis of chemiluminescence intensities in mice administered Cy5-labeled bacterial formulations 24 h after EcN or ECA@EcN administration (n = 3). (C, D) Chemiluminescence images and ROI analysis of intestinal tract chemiluminescence intensities in mice 24 h after EcN or ECA@EcN administration (n = 3). Data are presented as the means ± SD. Statistical analysis was conducted using Student’s t-test, *P < 0.05.


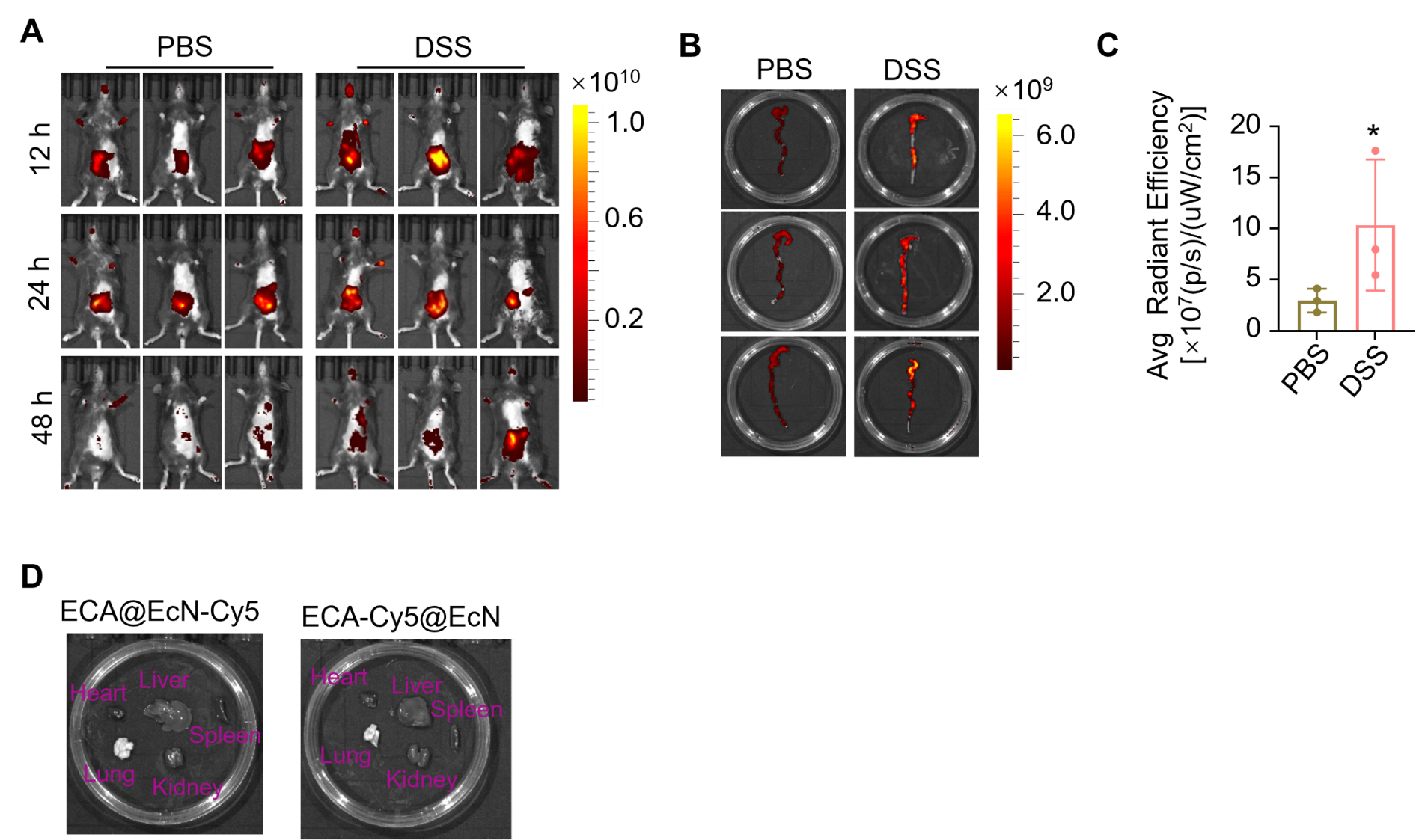


**Figure S17.** The fate of ECA@EcN in colon tissue. (A) Chemiluminescence images of normal and DSS-induced mice after administration of Cy5-labeled ECA@EcN at various time points. (B) Chemiluminescence images of intestinal tracts in mice 48 h post-administration of Cy5-labeled ECA@EcN. (C) Region-of-interest (ROI) analysis of chemiluminescence intensities in intestinal tracts 48 h post-administration (n = 3). (D) Distribution of Cy5-labeled EcN and ECA in major organs after ECA@EcN treatment for 48 h. Statistical analysis was conducted using Student’s t-test. *P < 0.05.


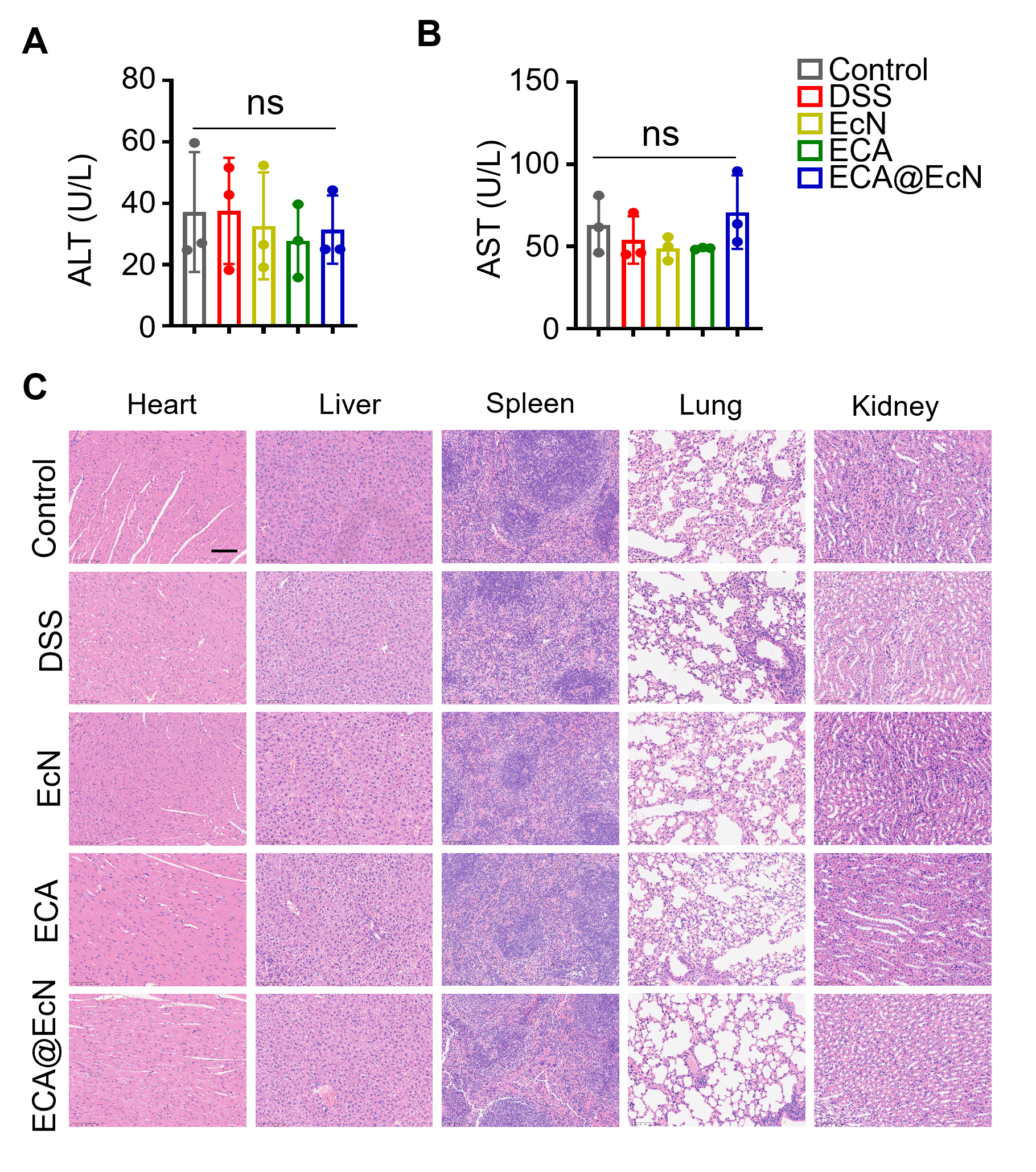


**Figure S18.** Safety profile of ECA@EcN in the preventive model. (A, B) Serum biomarker levels of AST and ALT were analyzed to assess hepatotoxicity. (C) H&E-stained sections of major organs (heart, liver, spleen, lung, and kidney) were analyzed for systemic toxicity. Scale bar: 100 µm. Data are presented as the means ± SD. Statistical analysis was conducted using one-way ANOVA. *P < 0.05; ns, not significant.


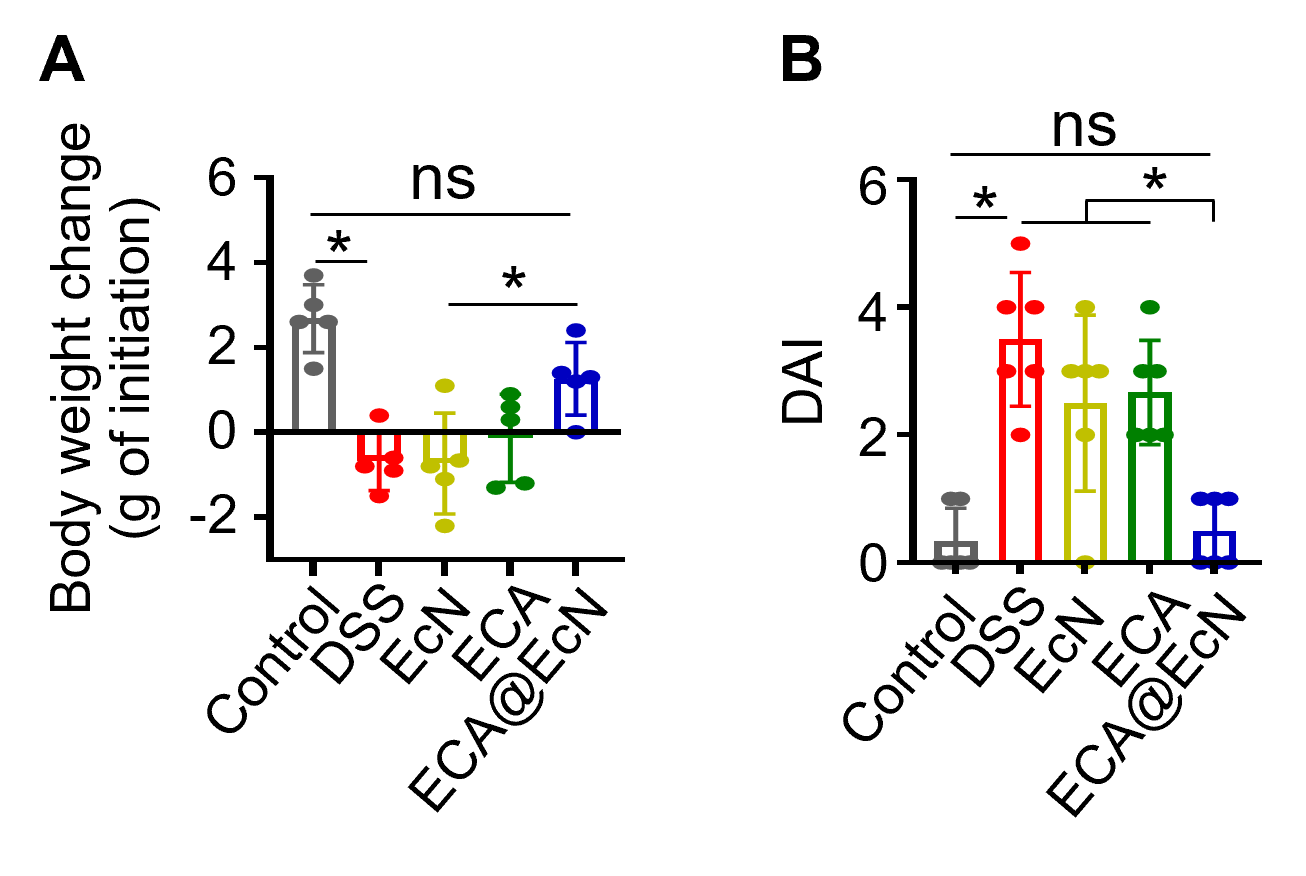


**Figure S19.** Body weight (A) and DAI (B) of the mice following different treatments. Data are presented as the means ± SD. Statistical analysis was conducted using one-way ANOVA. *P < 0.05; ns, not significant.


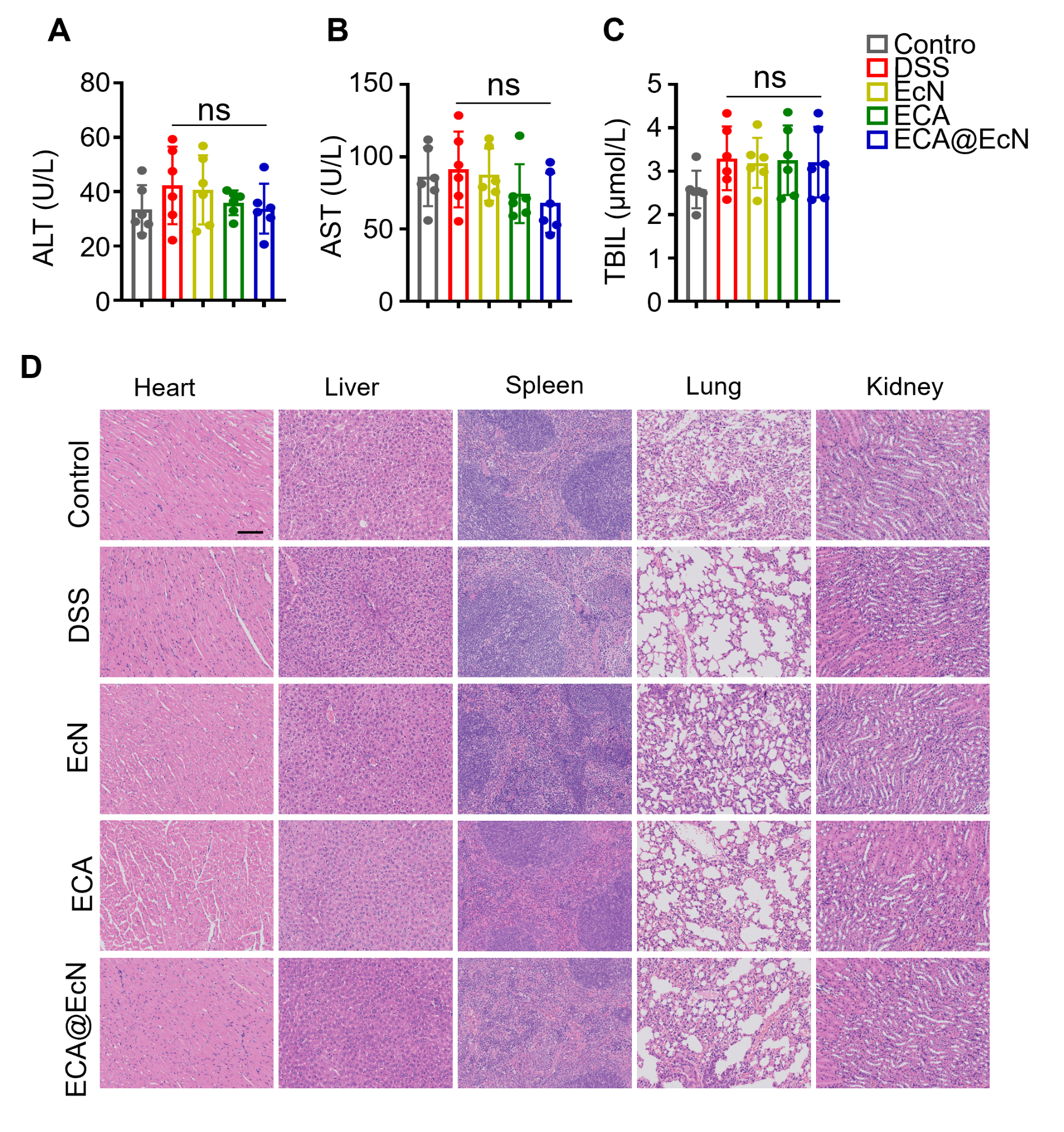


**Figure S20.** Safety profile of ECA@EcN in the therapeutic model. (A-C) Serum biomarker levels of AST, ALT, and TBIL were analyzed to assess hepatotoxicity. (D) H&E-stained sections of major organs (heart, liver, spleen, lung, and kidney) were analyzed for systemic toxicity. Scale bar: 100 µm. Data are presented as the means ± SD. Statistical analysis was conducted using one-way ANOVA. *P < 0.05; ns, not significant.


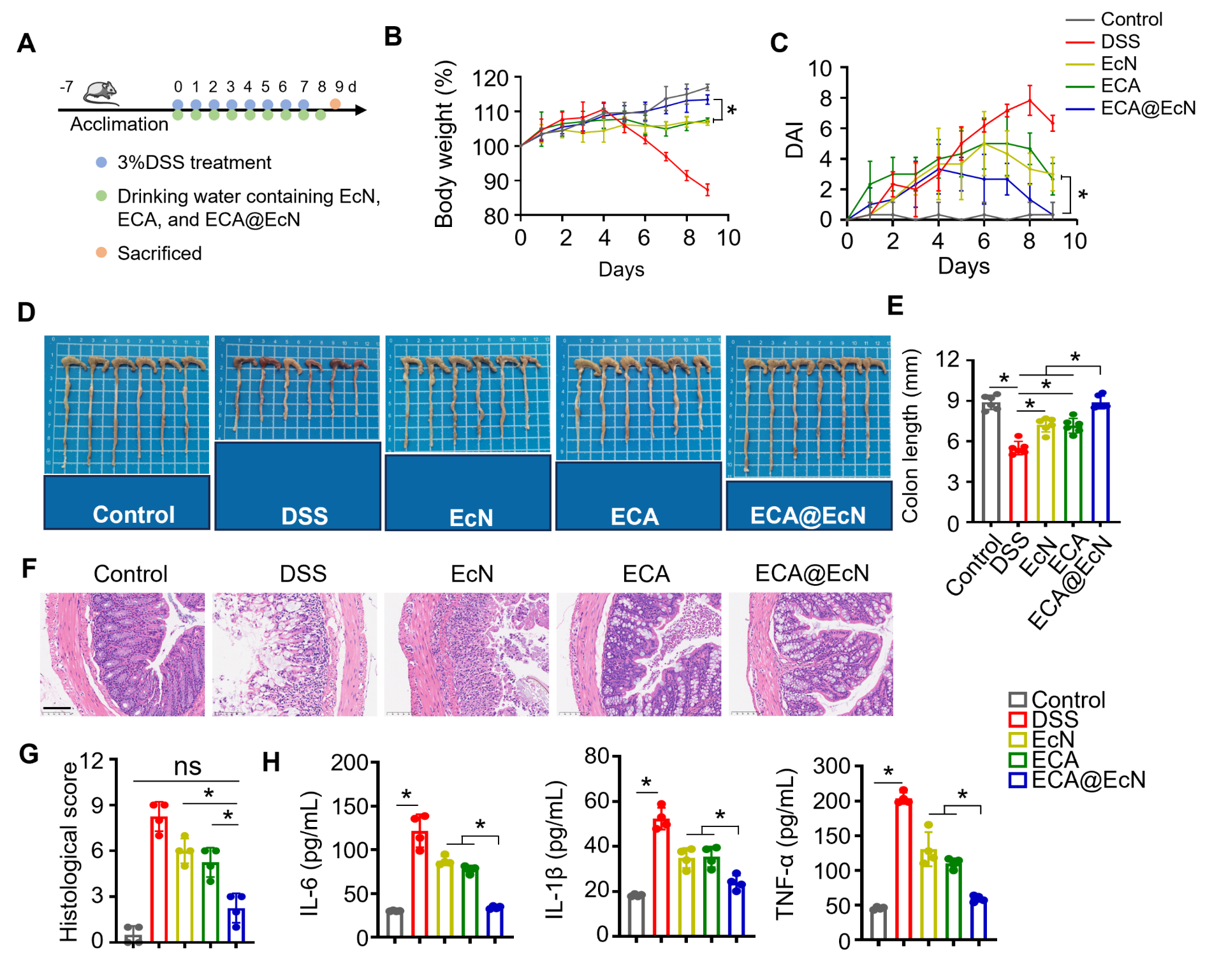


**Figure S21.** Preventive efficacy of ECA@EcN against DSS-induced colitis in mice. (A) Schematic showing the administration schedule in the DSS-induced acute colitis model. (B) Body weight changes in mice following different treatments (n = 6). (C) Disease activity index (DAI) changes during treatment (n = 6). (D) Colon tissue images and (E) quantified colon lengths after different treatments (n = 6). (F) Representative images of colon tissues stained with H&E, and (G) histopathology scores based on H&E images (n = 4). (H) Serum levels of TNF-α, IL-6, and IL-1β measured by ELISA (n = 4). Scale bar: 100 μm. All data are presented as the means ± SD. Statistical analysis was conducted using one-way ANOVA. *P < 0.05; ns, not significant.
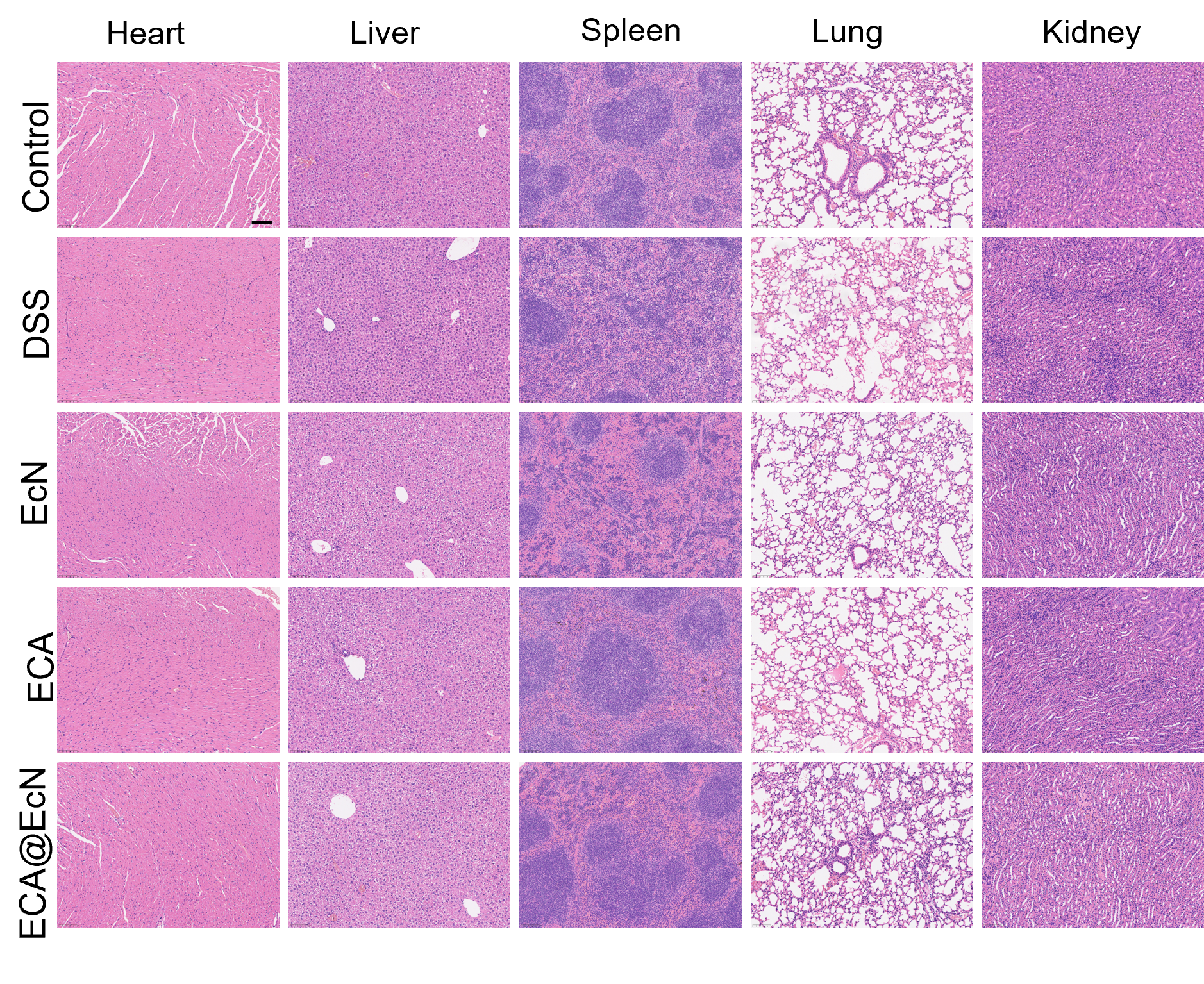


**Figure S22.** Representative H&E-stained images of heart, liver, spleen, lung, and kidney tissues. Scale bar: 100 μm.


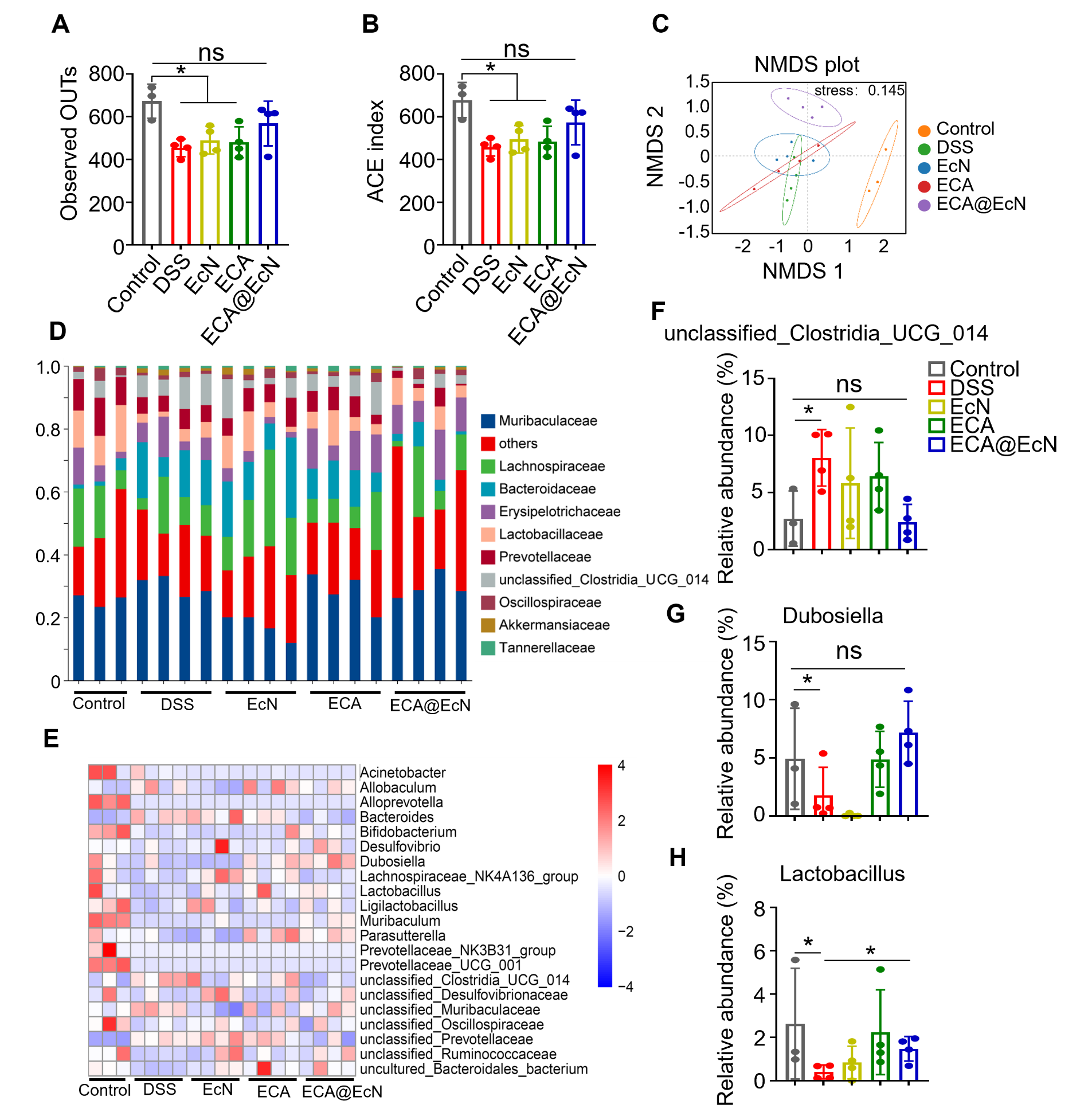


**Figure S23.** ECA@EcN modulates intestinal flora during IBD prevention. (A) Observed operational taxonomic unit (OTU) richness of the gut microbiota in mice following different treatments. (B) Gut microbiome α-diversity analyzed using the ACE index. (C) β-diversity of the fecal microbiome visualized via a non-metric multidimensional scaling (NMDS) plot. (D) Heatmap illustrating the relative abundance of gut microbes at the genus level across different treatment groups. (E) Relative abundance of the gut microbiome at the family level across different treatment groups. (F–H) Relative abundances of unclassified_Clostridia_UCG_014 (F), Dubosiella (G), and Lactobacillus (H) at the genus level in the gut microbiome of mice across different treatment groups. Sample sizes (n = 3 or 4). Data are presented as the means ± SD. Statistical analysis was conducted using one-way ANOVA (A, B) or Student’s t-test (F–H). *P < 0.05; ns, not significant.

**Table S1.** Primers used in this study

| **Primers** | **Forward Primer** | **Reversed Primer** |
| --- | --- | --- |
| β-actin | 5'-GGTGTGATGGTGGGAATGGG-3' | 5'-ACGGTTGGCCTTAGGGTTCAG-3' |
| IL-6 | 5'-CAACGATGATGCACTTGCAGA-3' | 5'-TGTGACTCCAGCTTATCTCTTGG-3' |
| IL-1β | 5'-TTCAAGGGGACATTAGGCAG-3' | 5'-TGTGCTGGTGCTTCATTCAT-3' |
| TNF-α | 5'-CTCAGCGAGGACAGCAAGG-3' | 5'-AGGGACAGAACCTGCCTGG-3' |
